# ApoM maintains cellular homeostasis between mitophagy and apoptosis by affecting the stability of *Nnt* mRNA through the Zic3-ApoM-Elavl2-Nnt axis during neural tube closure

**DOI:** 10.1038/s41419-025-07343-3

**Published:** 2025-01-19

**Authors:** Qing Liu, Dan Liu, Yuejiao Wang, Xiaowei Wei, Wei Ma, Hui Gu, Shanshan Jia, Yiwen He, Wenting Luo, Songying Cao, Zhonghua Yang, Anhua Wu, Zhengwei Yuan

**Affiliations:** 1 Key Laboratory of Health Ministry for Congenital Malformation, Shengjing Hospital, China Medical University, Shenyang, China; 2 Rheumatology and Immunology Department, Shengjing Hospital, China Medical University, Shenyang, China; 3 Department of Pediatric Surgery, Shengjing Hospital, China Medical University, Shenyang, China; 4 Department of Neurosurgery, Shengjing Hospital, China Medical University, Shenyang, China

**Keywords:** Disease model, Neurulation, Transcription, Mechanisms of disease, Apoptosis

## Abstract

Research on the aetiology of neural tube defects (NTDs) has made progress in recent years. However, the molecular mechanism of apolipoproteins underlying NTDs development remains unclear. This study aimed to investigate the function of apolipoprotein M (ApoM) in the pathogenesis of NTDs and its underlying mechanisms. We demonstrated that ApoM expression was reduced in the spinal cord samples of rat models and human fetuses with NTDs respectively. Specifically, lack of ApoM resulted in reduced cytosolic localization of Elavl2 and caused *Nnt* mRNA degradation, which further led to impaired cell homeostasis by suppressing PINK1-PRKN-mediated mitophagy and promoting apoptosis and subsequent NTDs formation. Moreover, Zic3 directly interacted with the promoter of ApoM and activated its transcription. Lastly, intra-amniotic delivery of adenoviral recombinant Zic3 or ApoM could promote mitophagy and alleviate apoptosis in spinal cords of NTDs. Collectively, these findings highlight the important role of the Zic3-ApoM-Elavl2-Nnt axis in cellular homeostasis during neural tube development, thereby revealing an intracellular molecular regulatory mechanism of ApoM, providing a mechanistic basis for understanding embryonic neural development, and offering experimental evidence for potential therapeutic targets for NTDs.

## Introduction

Neural tube defects (NTDs) are severe congenital malformations of the nervous system, with a global prevalence of 1.86‰ in 2015 [1]. Occurring during the first 21–28 days of pregnancy, NTDs involve failed fusion of neural folds in the midline [2]. An inadequate understanding of their pathogenesis hampers efforts to treat NTDs [3, 4]. Neural fold fusion depends on precise spatiotemporal expression of multiple genes; approximately 400 NTD-related genes have been found to date [5, 6]. Thus, multiple genetic, environmental and nutritional factors are considered to influence NTD etiology [1, 7–9].

Apolipoproteins may seem to be involved in NTDs, the exact relationship is unclear. Notably, ApoC1 is involved in neural boundary formation [10]. ApoB mutations in humans and mice cause NTDs [11–15]. Furthermore, we previously demonstrated that ApoA4 is downregulated in the amniotic fluid of NTD mice [16]. Another well-researched apolipoprotein is ApoM, implicated in HDL, cholesterol, sphingosine-1-phosphate (S1P), and lipid metabolism. Using iTRAQ quantitative proteomics, we found ApoM decreased in the sera of pregnant NTD rats [17]. Hyperlipidaemia and diabetes are proven risk factors for NTDs [18, 19]; ApoM is associated with both conditions, as well as with atherosclerosis and cardiovascular disease [20–22].

To date, research on ApoM have primarily focused on its role as a chaperone of S1P, a bioactive lipid [23]. ApoM forms a complex with S1P and transports the latter to its receptors on recipient cells, activates downstream signalling pathways [24, 25] to regulate physiological and pathological processes [26–28]. In addition to extracellular signaling, ApoM has intracellular signaling functions. It is a transcriptional regulator of LRH-1 [29], as well as a participant in assembly and release of hepatitis C virus. Specifically, ApoM interacts with the E2 protein of hepatitis C virus and binds to nascent virus particles [30]. However, whether ApoM is involved in NTD pathogenesis through intracellular regulatory mechanisms remains unclear.

Herein, we demonstrated for the first time that the ApoM deficiency in NTDs affects Elavl2 subcellular localization and disruptes *Nnt* mRNA stability. Disruption of the Zic3-ApoM-Elavl2-Nnt axis impairs cellular homeostasis via suppressing mitophagy and promoting apoptosis. These processes eventually lead to NTD formation.

## Materials and methods

### Human fetal spinal cord samples

Six fetuses with spina bifida at 24–33 weeks of pregnancy and 6 normal fetuses were selected from the Shengjing Birth Cohort. All fetuses were matched by maternal and gestational ages. The study is an ongoing prospective cohort study that has enrolled pregnant women, spouses, and their children living in Northeastern China since April 2017. Gynaecologists and fetal ultrasonologists at our hospital evaluated the classification of malformations and ensured the accuracy of the final diagnosis. Spinal cord samples from spina bifida fetuses were obtained from the edge of the defect area. The control samples were obtained from corresponding segments of normal spinal cord (approval no. 2017PS264K).

### Animal models

Outbred Wistar rats (10–12 weeks old, weighing 240–300 g each) were provided by the Animal Center of Shengjing Hospital, China Medical University (Shenyang, Liaoning, China). The female rats were mated with the males overnight. The morning during which the sperm were observed under a microscope was considered gestational day 0 (E0). Pregnant rats were induced by a single intragastric administration of ATRA (4% [wt/vol] in olive oil; 140 mg/kg body weight; Sigma-Aldrich, St. Louis, MO, USA) or olive oil on E10. The posterior spinal cords of embryos were dissected on E11, E12 and E14 and stored at −80 °C for further analysis. All animal experiments were conducted in accordance with the guidelines of the Medical Ethics Committee of the Shengjing Hospital of China Medical University (approval no. 2020PS153K).

### Small interfering RNA (siRNA) and plasmid construction

The siRNAs against ApoM, Nnt and Zic3 were synthesised using RIBOBIP (Guangzhou, China). The sequences of the siRNAs were as follows: ApoM siRNA 5′-CCTCTTGCTTGGACTTCAA-3′; Nnt siRNA 5′-GCTTACCTTGGCACTTACA-3′; and Zic3 siRNA 5′-GAACAACCACGTCTGCTAT-3′. ApoM, Nnt, Zic3 and Elavl2 overexpression plasmids and the control plasmid were purchased from SyngenTech Inc. (Beijing, China).

### Cell culture and transfection

C17.2 mouse neural stem cell line purchased from Beina Chuanglian Biology Research Institute were cultured in minimum essential medium (MEM; Gibco, MA, USA) with 10% foetal bovine serum (FBS; Gibco, MA, USA) and 1% MEM with non-essential amino acids (Gibco, MA, USA). All cells were maintained in a humidified 37 °C incubator with 5% CO_2_. The transfections were performed using Lipofectamine 3000 (Invitrogen, Waltham, MA, USA) according to the manufacturer’s protocol. After specific times of transfection, cells were harvested and stored at −80 °C prior to further analyses.

### RNA extraction and real-time quantitative reverse-transcription PCR (qRT-qPCR)

Total RNA was isolated from rat embryonic spinal cords and C17.2 cells using TRIzol reagent (Takara, Ohtsu, Japan) in accordance with the manufacturer’s recommendations. An ultra-micro spectrophotometre (Thermo Scientific Nanodrop 2000, Waltham, MA, USA) was used to measure the total RNA concentration and purity. Genomic DNA was removed, and RNA was reverse-transcribed into cDNA using a PrimeScript RT reagent kit (Takara, Ohtsu, Japan). mRNA expression levels were measured using the SYBR Premix Ex Taq kit (Takara, Ohtsu, Japan) on a 7500 Real-Time PCR System (StepOnePlus, ABI Company, Oyster Bay, NY, USA). Primers were designed and synthesised by Sangon Biotech (Shanghai, China) and are listed in Supplementary Table S3. The classic 2^−^^ΔΔCt^ method was used to analyse relative gene expression after normalisation to GAPDH.

### Protein extraction and immunoblotting

Rat embryonic spinal cords or C17.2 cells were lysed in radioimmunoprecipitation buffer (Solarbio Science & Technology, Beijing, China) to obtain the total protein. Minute^TM^ Mitochondrial Extraction kits (Invent Biotechnologies, Inc., Plymouth, MN, USA) were used to isolate the mitochondrial and cytosolic fractions, following the manufacturer’s instructions. Nuclear Protein Extraction kits (Abmart, #A10009, Shanghai, China) were used to extract the nuclear and cytoplasmic proteins according to the manufacturer’s instructions. Protein concentrations were determined using bicinchoninic acid assays (Takara, Ohtsu, Japan).

Equal amounts of protein were separated by sodium dodecyl sulphate-polyacrylamide gel electrophoresis and electrophoretically transferred to polyvinylidene difluoride membranes (Millipore, MA, USA). The membranes were blocked with 5% nonfat milk solution for 1 h and subsequently incubated with primary antibodies overnight at 4 °C. The following primary antibodies were used in the experiment: anti-ApoM (1:1000, Cell Signaling Technology, #5709, MA, USA), anti-Nnt (1:1000, Proteintech, 13442-2-AP, Wuhan, China), anti-Cytochrome c (1:1000, Cell Signaling Technology, #11940, MA, USA), anti-AIF (1:1000, Cell Signaling Technology, #5318, MA, USA), anti-VDAC1 (1:2000, Proteintech, 55259-1-AP, Wuhan, China), anti-Bcl2 (1:1000, Proteintech, 26593-1-AP, Wuhan, China), anti-Bax (1:1000, Cell Signaling Technology, #14796, MA, USA), anti-Caspase3 (1:1000, Novus Biologicals, NB100-56708, CO, USA), anti-Caspase9 (1:1000, Proteintech, 10380-1-AP, Wuhan, China), anti-PINK1 (1:1000, Proteintech, 23274-1-AP, Wuhan, China), anti-PRKN (1:2000, Proteintech, 14060-1-AP, Wuhan, China), anti-LC3 (1:1000, Cell Signaling Technology, #4108, MA, USA), anti-Elavl2 (1:3000, Proteintech, 14008-1-AP, Wuhan, China), anti-Myc (1:1000, Proteintech, 16286-1-AP, Wuhan, China), anti-Lamin B1 (1:50000, Proteintech, 66095-1-Ig, Wuhan, China), anti-Zic3 (1:1000, Novus Biologicals, NBP1-33207, CO, USA), and anti-GAPDH (1:5000, Proteintech, 60004-1-Ig, Wuhan, China). After incubation with the corresponding secondary antibody (1:5000, Proteintech, Wuhan, China), the protein bands were detected using a chemiluminescent substrate (Millipore, MA, USA) and quantified using ImageJ 1.8.0 software (National Institutes of Health, Bethesda, MD, USA).

### Mitochondrial permeability transition pore (mPTP) opening assay

The opening of mPTP was assessed using the cobalt quenching of calcein-AM fluorescence (Beyotime, C2009S, Shanghai, China). After being transfected with ApoM-siRNA, C17.2 cells were treated with or without carbonyl cyanide m-chlorophenylhydrazine (CCCP, Beyotime, Shanghai, China) for 30 min according to the manufacturer’s protocol and harvested using trypsin and loaded with calcein-AM (1 μM) at 37 °C for 30 min in the dark. CoCl2 (1 mM) was then added, and the cells were incubated for another 30 min. The fluorescence of the cells in each experiment was measured using a Tecan Infinite 200 Pro Microplate reader (Tecan, NSW, Australia). For the imaging experiments, the cells were cultured in 12-well plates and treated according to the protocol described above after transfection. Cell images were captured using an ECLIPSE 80i fluorescence microscope (Nikon, Kyoto, Japan). Data were analysed using ImageJ software.

### Determination of reactive oxygen species (ROS)

A Reactive Oxygen Species Assay kit (Beyotime, S0033, Shanghai, China) was used to determine the ROS levels. After being transfected, C17.2 cells were treated with or without hoxidant Rosup (Beyotime, Shanghai, China) for 30 min according to the manufacturer’s protocol and harvested using trypsin and resuspended in MEM without FBS. The cells were then incubated with DCFH-DA (diluted 1:1000) in a 37 °C incubator in the dark for 30 min. We used a Tecan Infinite 200 Pro Microplate reader (Tecan, NSW, Australia) to measure the fluorescence intensity of DCFH and set the excitation and emission wavelengths to 488 and 525 nm, respectively. For the imaging experiments, the cells were cultured in 12-well plates and observed under an ECLIPSE 80i fluorescence microscope (Nikon, Kyoto, Japan). The data were analysed using ImageJ software.

### Detection of mitochondrial membrane potential

The abundance of mitochondrial membrane potential was measured using a JC-1 fluorescent probe (Beyotime, C2006, Shanghai, China). The transfected C17.2 cells were treated with or without CCCP for 30 min according to the manufacturer’s protocol and collected using trypsin and incubated with JC-1 working solution at 37 °C for 30 min. The abundance of mitochondrial membrane potential was determined using a Tecan Infinite 200 Pro Microplate reader (Tecan, NSW, Australia). For the imaging experiments, the cells were cultured in 12-well plates and treated according to the protocol described above after transfection. The cells were observed under a laser scanning confocal microscope (Zeiss, Oberkochen, Germany), and the data were analysed using ImageJ software.

### Transmission electron microscopy

C17.2 cells were seeded onto 10-centimetre plates and transfected with ApoM siRNA for 48 h. The cells were treated with CCCP for the last 12 h. The cells were darkly fixed with 2.5% glutaraldehyde at room temperature and then dehydrated, infiltrated and embedded for analysis. Ultra-thin sections were collected using ultramicrotome (Leica Microsystems, EM UC7, Wetzlar, Germany) and observed using transmission electron microscopy (Hitachi High-Tech Co., HT 7800, Tokyo, Japan).

### RNA stability measurement

*Nnt* mRNA stability was measured using an actinomycin D (Act D, Sigma-Aldrich, MO, USA) assay. In brief, 20 µg/mL of Act D was added into the cell media at 48-h post-transfection with siRNAs or plasmids. After 0, 1, 2, 4 and 8 h of the Act D treatment, RNA was isolated from the cells, and the *Nnt* mRNA levels were tested using qRT-PCR. The ‘relative one phase decay model’ of nonlinear regression was used to fit the RNA expression value decay curve over time and calculate the half-life time (*t*_1/2_).

### Co-immunoprecipitation (Co-IP)

C17.2 cells were subjected to endogenous Co-IP assay and cells transfected with overexpression of Elavl2-Myc plasmid were subjected to exogenous Co-IP assay. Cells were lysed in IP/western lysing solution (Beyotime, P0013, Shanghai, China) for 60 min on ice and then centrifuged at 12,000 × *g* for 15 min to remove debris. For immunoprecipitation, 4 µg of ApoM antibody (Cell Signaling Technology, #5709, MA, USA), Elavl2 antibody (Proteintech, 14008-1-AP, Wuhan, China) or Myc antibody (Proteintech, 16286-1-AP, Wuhan, China) was bound to Protein G Magnetic Beads (Cell Signaling Technology, #70024, MA, USA) on a rotator at room temperature for 45 min, with the corresponding IgG (Cell Signaling Technology, MA, USA) used as a negative control. Lysates were incubated with the antibody-crosslinked resin overnight at 4 °C to accomplish antigen immunoprecipitation and analysed by immunoblotting.

### RNA immunoprecipitation-qPCR assay (RIP)

The RIP assay was performed using the EZ-Magna RIP RNA-binding protein immunoprecipitation kit (Millipore, 17–700, MA, USA) according to the manufacturer’s recommendations. The cells (1.0 × 10^7^) were washed with cold PBS and lysed on ice using the harsh lysis buffer. The supernatants were collected by centrifugation at 15,000 × *g* and 4 °C. Magnetic beads pre-incubated with IgG or antibodies specific to ApoM (Cell Signaling Technology, #5709, MA, USA) or Elavl2 (Cell Signaling Technology, #5709, MA, USA) were incubated with lysates overnight at 4 °C. An aliquot of the lysate was used as the input control. Immunoprecipitated and input RNAs were isolated and analysed by qRT-PCR. The immunoprecipitated *Nnt* mRNA was normalised to the input control. The primers used are listed in Supplementary Table S3.

### RNA pull-down assay

An RNA pull-down assay was performed using an RNA pull-down kit (Bersin Bio, Guangzhou, China) according to the manufacturer’s protocol. Biotin-labelled Nnt and LacZ probes were constructed by Biosense Bioscience Co., Ltd. (Guangzhou, China). Briefly, biotin-labelled RNAs were captured by streptavidin-conjugated magnetic beads for 2 h at 25 °C. Next, the complex comprising biotin-labelled RNA and lysates of C17.2 cells was purified using streptavidin-agarose for 2 h at 4 °C. Finally, the RNA-conjugated proteins were eluted, and the protein level of Elavl2 was determined by western blotting.

### Fluorescence in situ hybridisation (FISH) and immunofluorescence (IF) staining

FISH probes and a FISH kit were provided by RiboBio (Guangzhou, China). C17.2 cells were seeded onto coverslips in 24-well plates and transfected for 48 h. The cells were then fixed with 4% paraformaldehyde for 30 min and permeabilised with 0.5% Triton X-100 for 30 min. The cells were washed thrice with PBS and treated with pre-hybridisation buffer containing a 1% blocking solution. The Cy3-labelled Nnt probe was diluted in hybridisation buffer containing a 1% blocking solution (100 µL). The surface of the coverslip was covered for incubation at 37 °C for 12 h in a dark and humid chamber. Next, the cells were washed five times with gradient standard saline citrate at 42 °C and treated with Elavl2 antibody (1:200, Proteintech, 14008-1-AP, Wuhan, China) at 4 °C overnight, followed by incubation with Alexa Fluor 488 (1:500, Cell Signaling Technology, #4412, MA, USA) for 2 h at room temperature in the dark. Nuclei were counterstained with 4′,6-diamidino-2-phenylindole (DAPI), and images were acquired using a laser scanning confocal microscope (Zeiss, Oberkochen, Germany).

### Chromatin immunoprecipitation-qPCR assay (ChIP)

A ChIP assay was conducted using the SimpleChIP Enzymatic Chromatin IP Kit (Cell Signaling Technology, #9002, MA, USA), following the manufacturer’s instructions. Briefly, C17.2 cells were cultured until there were approximately 1 × 10^7^ cells and cross-linked with 1% formaldehyde. Samples were then harvested, and chromatin was digested with micrococcal nuclease and sonicated to a length of approximately 150–900 bp. An aliquot of each ChIP sample was prepared as an input control, and the rest of the chromatin was immunoprecipitated with anti-Zic3 (Novus Biologicals, NBP1-33207, CO, USA) or anti-IgG as a negative control overnight at 4 °C. After being washed and reverse cross-linked, the eluted DNA was purified using columns and quantified by qPCR. The precipitated genomic DNA was amplified using the primers for the ApoM promoter region listed in Supplementary Table S3.

### Luciferase reporter assay

The pGL4.18 plasmid containing the wild-type (WT) promoter sequence of *Nnt* mRNA (Nnt-WT) was cotransfected into C17.2 cells with or without the ApoM plasmid. Another pGL4.18 plasmid containing the WT (ApoM-WT) or mutant-type (ApoM-Mut) promoter sequences of *ApoM* mRNA was cotransfected into C17.2 cells with or without the Zic3 plasmid. Luciferase reporter plasmids were cotransfected into C17.2 cells. After 48 h, luciferase activity was detected using a Dual-Luciferase Reporter Assay System (Promega, WI, USA) and measured using a Tecan Infinite 200 Pro Microplate reader (Tecan, NSW, Australia). Relative luciferase activity was calculated as the ratio of firefly to Renilla luciferase activity.

### Recombinant adenoviruses and intra-amniotic injections

GFP, rat Zic3 cDNA and rat ApoM cDNA were cloned into E1- and E3-deleted human Ad type 5 vectors under the control of cytomegalovirus promoter, generating recombinant Ad (i.e., Ad-GFP, Ad-GFP-Zic3 or Ad-GFP-ApoM). The viral titres were 2.51 × 10^11^ pfu/mL for Ad-GFP-Zic3, 1.99 × 10^11^ pfu/mL for Ad-GFP-ApoM, and 2.51 × 10^11^ pfu/mL for Ad-GFP. The adenoviruses used in this study were obtained from Hanbio Biotechnology (Shanghai, China). Intra-amniotic Ad injections were administered to E16 embryos as previously described [31, 32]. Pregnant rats were anaesthetised with pentobarbitone sodium (40 mg/kg of body weight). An incision was made in the abdominal wall, and the uterus was exteriorised. Under an operating microscope, fetuses with a uniform position and defective-sized spina bifida were chosen and randomly divided into PBS-, Ad-GFP-, Ad-GFP-Zic3- and Ad-GFP-ApoM-injected groups. Microinjections were performed with transuterine injections of 5 μL of solution using a glass micropipette (internal tip diameter, 100 μm) connected to a Hamilton syringe. The micropipettes for the injections were made from borosilicate glass capillaries (model GD-1; Narishige Scientific Instruments, Tokyo, Japan) with a micropipette puller (model PB-7; Narishige Scientific Instruments). After the injection, the uterus was returned to the abdomen, and the abdominal wall was closed. The pregnant rats recovered from anaesthesia within 1 h and were returned to their home cages. They were euthanised on E21 with an overdose of pentobarbitone sodium, and the injected fetuses were harvested for analysis.

### Fluorescence imaging and tissue preparation

On E21, the injected fetuses were harvested, and a fluorescence stereomicroscope (Leica, M165FC, Germany) fitted with a Nikon DS-Qi2 digital camera (Nikon, NY-1S35, Japan) was used to capture in vivo fluorescence images of the fetuses. The fetuses were then transcardially perfused with 15 mL of physiological saline, followed by 25 mL of 4% paraformaldehyde. The lumbosacral spinal column containing muscle, spinal cord, and subcutaneous tissue was dissected and post-fixed in 4% paraformaldehyde overnight. The tissues were then cryoprotected in 20% sucrose for 24 h, embedded with the optimal cutting temperature compound (SAKURA, Japan), and sectioned into 30-micrometre-thick slices using a freezing microtome (Thermo, Microm HM525, Germany). GFP-positive cells were observed using an ECLIPSE 80i fluorescence microscope (Nikon, Kyoto, Japan). All of the sections were stored at −80 °C for subsequent experiments.

### TUNEL staining

The TUNEL assay was performed using the One Step TUNEL Apoptosis Assay Kit (Beyotime, C1090, Shanghai, China). Based on the manufacturer’s protocol, the sections were fixed with 4% paraformaldehyde for 1 h and 0.5% Triton x-100 for 5 min at room temperature. The sections were then washed and incubated with TUNEL reaction mixture at 37 °C for 1 h in the dark. After being washed, the sections were incubated for 5 min with DAPI (Beyotime, C1002, Shanghai, China) and mounted using anti-fade mounting media. Images were acquired using an ECLIPSE 80i fluorescence microscope (Nikon, Tokyo, Japan).

### IF staining

C17.2 cells were seeded onto coverslips in 12-well plates and transfected for 48 h. The cells were then fixed with 4% paraformaldehyde and permeabilised with 0.5% Triton for 30 min. After being blocked with 5% bovine serum albumin (BSA) for 45 min, the coverslips were transferred to a humidified chamber and incubated with the primary antibodies mouse anti-Tomm20 (1:1000, Abcam, ab56783, MA, USA) and rabbit anti-LC3 (1:100, Cell Signaling Technology, #4108, MA, USA) overnight at 4 °C, followed by incubation with Alexa Fluor 488 (1:500, Cell Signaling Technology, #4412, MA, USA) and Alexa Fluor 555 (1:500, Cell Signaling Technology, #4409, MA, USA) for 2 h at room temperature in the dark.

The frozen sections were fixed with 4% paraformaldehyde and permeabilised with 0.5% Triton for 1 h. They were then blocked with 10% BSA for 1 h. The primary antibodies used were mouse anti-Tomm20 (1:1000, Abcam, ab56783, MA, USA) and rabbit anti-LC3 (1:100, Cell Signaling Technology, #4108, MA, USA), and the secondary antibodies included Alexa Fluor 647 (1:1000, Invitrogen, A32795, MA, USA) and Alexa Fluor 555 (1:500, Cell Signaling Technology, #4409, MA, USA). Nuclei were counterstained with DAPI, and images were acquired using a laser scanning confocal microscope (Zeiss, Oberkochen, Germany).

### Statistical analyses

Statistical analyses and graph generation were performed using GraphPad Prism 7.0 software (GraphPad Software). All data are presented as the mean ± standard deviation. A two-tailed Student’s *t* test was used to analyse the differences between two groups, and a one-way analysis of variance (ANOVA) was used to analyse the data among multiple groups. All the measurements were repeated at least three times, with consistent trends and differences, and the results were considered to be statistically significant at *P < 0.05 and **P < 0.01, with “ns” representing no significance.

## Results

### ApoM and Nnt are downregulated in the spinal cords of NTDs rat models and NTDs human fetuses

**Fig. 1:**
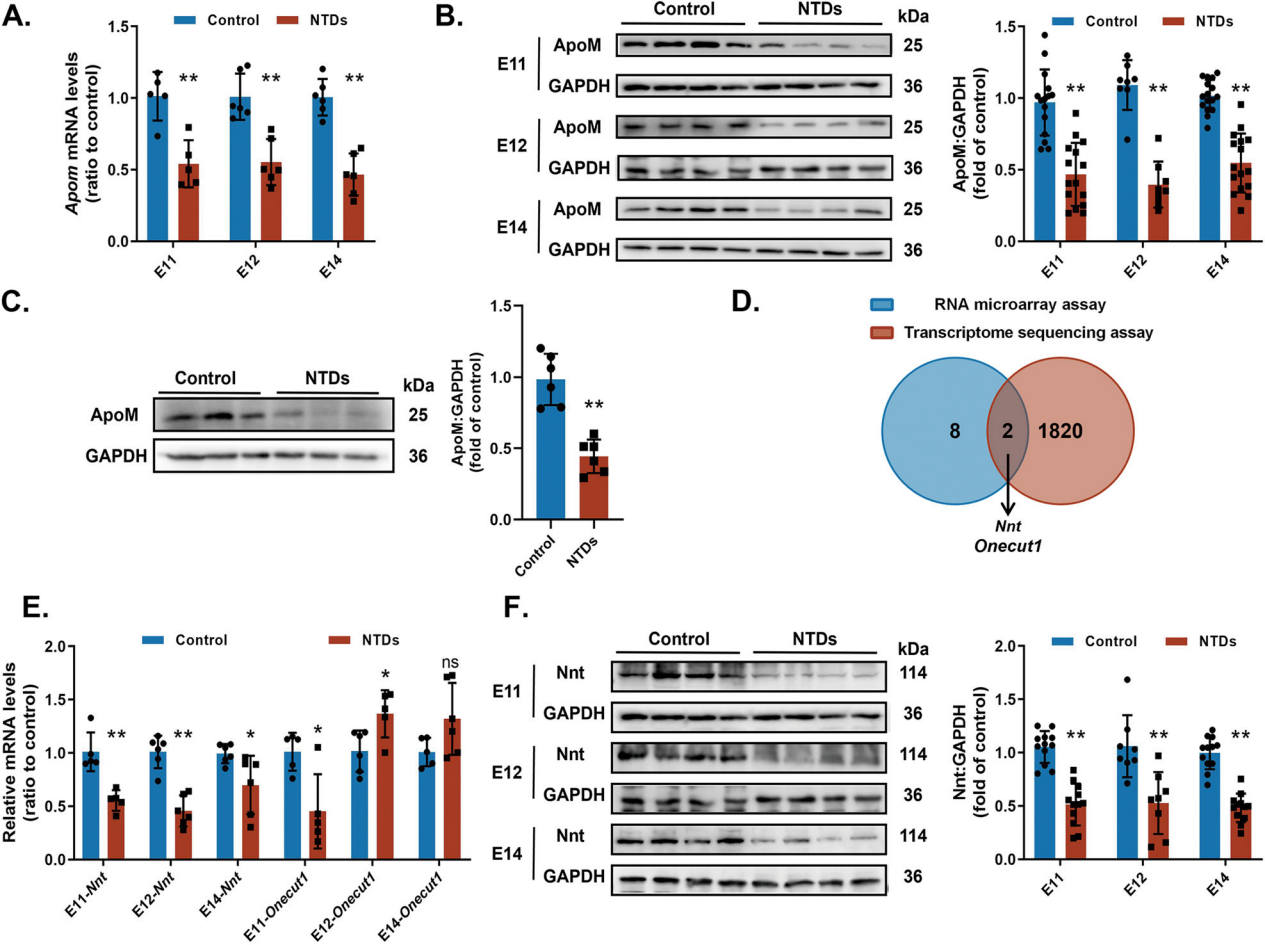
ApoM and Nnt are downregulated in rat models and human fetuses with NTDs. **A**, **B**, **E**, **F** Rat models were induced with a single intragastric administration of ATRA (NTDs) or olive oil (Control). Rat embryonic neural tube tissues were dissected on E11, E12 and E14. (**A** *ApoM* mRNA expressions in the Control and NTDs groups were assessed by qRT-PCR (*n* = 5–6 for each group). **B** ApoM protein expressions in the Control and NTDs groups were analysed by western blotting (*n* = 8 for each group). **C** ApoM protein expressions of human fetal spinal cords in the Control and NTDs groups were analysed by western blotting (*n* = 8 for each group). **D** Venn diagram of the top 10 differentially expressed genes (DEGs) of liver extracts between ApoM^−/−^ and wild-type (WT) mice, as determined by an RNA microarray assay vs. DEGs of rat embryonic spinal cords between the NTDs and Control groups on E11 and E12 identified by a transcriptomic sequencing assay. **E** *Nnt* and *Onecut1* mRNA expressions in the Control and NTDs groups were assessed by qRT-PCR (*n* = 5–6 for each group). **F** Nnt protein expressions in the Control and NTDs groups were analysed by western blotting (*n* = 8 for each group). GAPDH was used as a loading control. Data are expressed as the mean ± standard deviation (SD). Statistical comparisons were performed using Student’s *t* tests. *P < 0.05; **P < 0.01; “ns” represents no significance vs. the indicated group.

We investigated ApoM expression in the embryonic spinal cords of normal and NTD rats. Both qRT-PCR and western blot analysis revealed a decrease in ApoM of the NTDs group at different gestational ages (E11, E12, E14) (Fig. 1A, B). Subsequently, we verified ApoM expression using normal and NTDs human fetal spinal cords (see Supplementary Table 1 for patient information). Western blotting results showed that ApoM protein levels were lower in NTDs group than in the control (Fig. 1C). suggesting that ApoM may be associated with NTDs.

We further identifed ApoM target genes that are involved in neural tube closure. We investigated *Nnt* and *Onecut1* out of the top 10 differentially expressed genes (DEGs) in liver extracts of ApoM^−/−^ mice [33]. The two selected genes were also DEGs in the embryonic neural tube of NTDs rat models, based on our previous transcriptomic sequencing data (Fig. 1D) [34]. Results from qRT-PCR showed that *Nnt* mRNA levels were reduced in the NTDs group at E11, E12 and E14, indicating that Nnt may be a downstream target of ApoM. However, *Onecut1* mRNA levels remained unstable (Fig. 1E). Consistent with transcriptional data, Nnt protein levels were lower in the NTDs group at the same time points (Fig. 1F). Abnormal ApoM expression may be the cause of Nnt downregulation in ATRA-induced NTDs.

### Silencing ApoM induces mitochondrial damage

Nnt encodes an enzyme in the inner mitochondrial membrane that is closely associated with ROS detoxification. Mitochondrial damage from oxidative stress is an important factor in NTD formation. Because ApoM targets NTD, we investigated the role of ApoM in mitochondrial function.

**Fig. 2:**
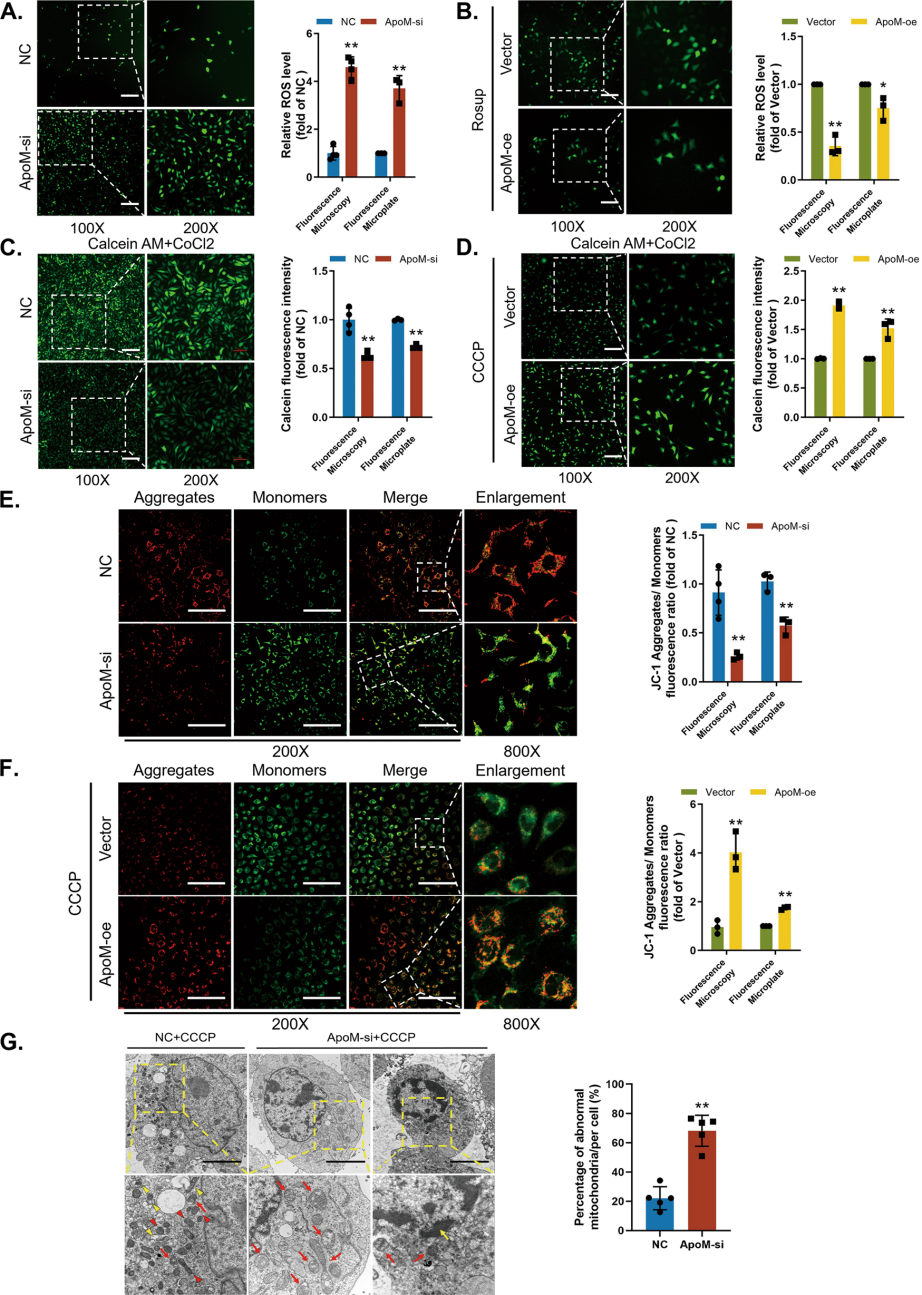
Absence of ApoM induces mitochondrial damage. **A**, **C**, **E**, **G** C17.2 cells were transfected with Ncontrol (NC) or siRNA targeting ApoM (ApoM-si). **B**, **D**, **F** C17.2 cells were transfected with empty pcDNA vector (Vector) or WT ApoM cDNA (ApoM-oe). C17.2 cells were treated with Rosup in (**B**) and with CCCP in (**D**, **F**, **G**). **A**, **B** Representative images and its quantification of ROS assay showed the intracellular ROS levels (*n* = 3 from independent experiments). Scale bar = 200 µm. **C**, **D** Representative images and its quantification of mPTP opening assay showed the changes in the fluorescent intensity of calcein (*n* = 3 from independent experiments). Scale bar = 200 µm. **E**, **F** Representative images and its quantification of JC-1 assay showed the changes in the fluorescence intensities of the JC-1 aggregates (red) and monomers (green) (*n* = 4 from independent experiments). Scale bar = 200 µm. **G** Mitochondrial morphology was assessed using transmission electron microscopy in C17.2 cells treated with CCCP. Red triangles, mitochondria with good morphology; yellow triangles, mitophagosomes; red arrows, cristae missing, swollen and vacuolated mitochondria; yellow arrows, apoptotic cell with shrunken cell body and smaller nuclei with massive chromatin condensation. Scale bar = 5 µm. Defective mitochondria rate = number of defective mitochondria divided by the total number of mitochondria (*n* = 5 from independent experiments). Statistical comparisons were performed using Student’s *t* tests. *P < 0.05; **P < 0.01; “ns” represents no significance vs. the indicated group.

After silencing ApoM at C17.2 cells, the ROS assay showed enhanced fluorescence intensity. Absorbance was also elevated, indicating that silencing ApoM induced ROS production (Fig. 2A). Conversely, ApoM overexpression reduced ROS production in cells treated with the oxidant Rosup (Fig. 2B). mPTP opening assay results demonstrated that, after ApoM silencing, excess ROS induced the opening of mPTP (Fig. 2C), leading to mitochondrial depolarization. In contrast, ApoM overexpression limited CCCP destruction of mPTP (Fig. 2D). We confirmed the occurrence of mitochondrial depolarization via JC-1 staining, after silencing ApoM, a diffuse green fluorescence replaced the red punctate representing normal mitochondria. The red/green fluorescence absorbance ratio also decreased (Fig. 2E). Overexpressing ApoM yielded the opposite result (Fig. 2F).

Mitochondrial morphology was observed under transmission electron microscopy (Fig. 2G). Silencing of ApoM increased mitochondrial damage (mitochondrial swelling, short cristae, and vacuolated matrix). Interestingly, ApoM silencing also reduced the number of mitophagosomes and promoted apoptosis (cell body shrinkage, smaller nuclei and massive chromatin condensation). In summary, abnormal ApoM expression causes mitochondrial dysfunction, in turn influencing mitophagy and apoptosis.

### Silencing ApoM affects cellular homeostasis through suppressing mitophagy and promoting apoptosis

**Fig. 3:**
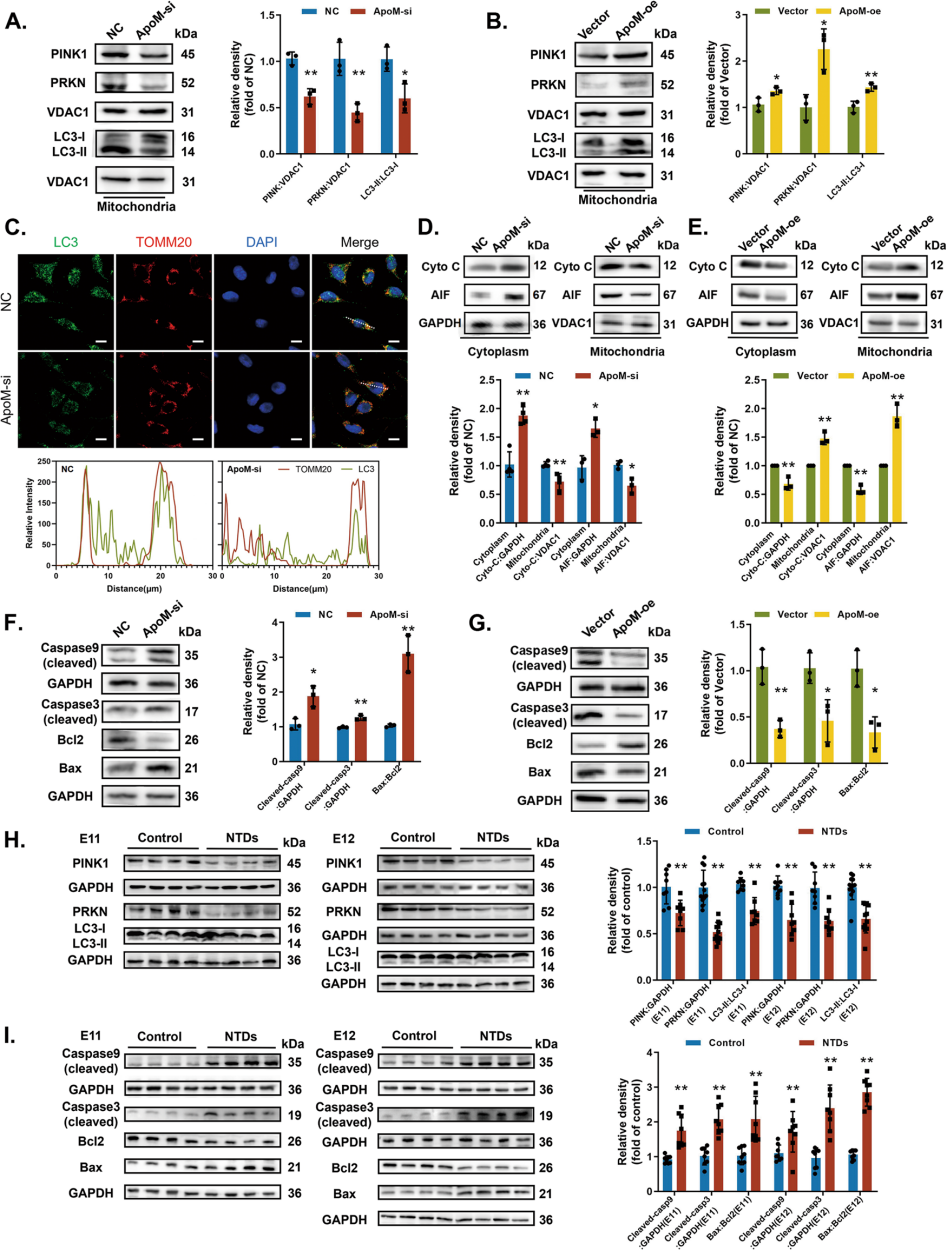
ApoM suppresses mitophagy and promotes apoptosis. **A**, **C**, **D**, **F** C17.2 cells were transfected with Ncontrol (NC) or siRNA targeting ApoM (ApoM-si). **B**, **E**, **G** C17.2 cells were transfected with empty pcDNA vector (Vector) or WT ApoM cDNA (ApoM-oe). **A**, **B** PINK1, PRKN and LC3-II/LC3-I protein levels in mitochondria were measured by western blotting (*n* = 3 from independent experiments). **C** C17.2 cells were double IF-stained with LC3-labelled autophagosomes (green) and TOMM20-labelled mitochondria (red). Nuclei were counterstained with DAPI. Scale bar = 10 µm. **D**, **E** Cytochrome c and AIF protein levels in the cytoplasm and mitochondria were measured by western blotting (*n* = 3 from independent experiments). **F**, **G** Cleaved Caspase 9, cleaved Caspase 3 and Bax/Bcl-2 protein levels were analysed by western blotting (*n* = 3 from independent experiments). **H**, **I** Rat models were induced with a single intragastric administration of ATRA (NTDs) or olive oil (Control). Rat embryonic neural tube tissues were dissected on E11 and E12. **H** PINK1, PRKN and LC3-II/LC3-I protein levels in the Control and NTDs groups were analysed by western blotting (*n* = 8 rats per group). **I** Cleaved Caspase 9, cleaved Caspase 3 and Bax/Bcl-2 protein levels in the Control and NTDs groups were analysed using western blotting (*n* = 8 rats per group). GAPDH or VDAC1 was used as a loading control. Data are expressed as the mean ± SD. Statistical comparisons were performed using Student’s *t* tests. *P < 0.05; **P < 0.01; “ns” represents no significance vs. the indicated group.

Mitophagy is an autophagic response that selectively removes excess or damaged mitochondria for mitochondrial quality control and cell homeostasis [35]. We confirmed the role of ApoM in mitophagy through measuring specific markers, namely PINK1 and PRKN expression and the mitochondrial LC3-II:LC3-I ratio. All markers decreased after ApoM silencing but increased after ApoM overexpression (Fig. 3A, B). We also observed low colocalization of LC3 and translocase of outer mitochondrial membrane 20 (TOMM20) after silencing ApoM, indicating the inhibition of mitophagy (Fig. 3C). After ApoM overexpression, colocalization of LC3 and TOMM20 rose considerably (Fig. S1). Therefore, ApoM appears to regulate PINK1-PRKN-mediated mitophagy.

When mitophagy is dysregulated, damaged mitochondria are not removed in time. As a result, mPTP remains open, leading to elevated release of apoptosis-inducing mitochondrial proteins. Two of these, cytochrome c and apoptosis-inducing factor (AIF), activate caspase- and non-caspase-mediated apoptotic pathways, respectively.

Here, western blotting showed that cytochrome c and AIF decreased in mitochondria and increased in cytoplasm after silencing ApoM (Fig. 3D). However, ApoM overexpression inhibited the release of both proteins (Fig. 3E). Silencing ApoM also upregulated pivotal apoptosis-related proteins, including Bax/Bcl2 and cleaved Caspases 9 and 3 (Fig. 3F); all were downregulated by ApoM overexpression (Fig. 3G). Moreover, mitophagy activation markers decreased significantly in NTDs rat models, while apoptosis-related proteins increased (Fig. 3H, I). Overall, these results suggest that part of NTDs etiology involves an imbalance between mitophagy and apoptosis.

### ApoM maintains cell homeostasis through acting on Nnt

**Fig. 4:**
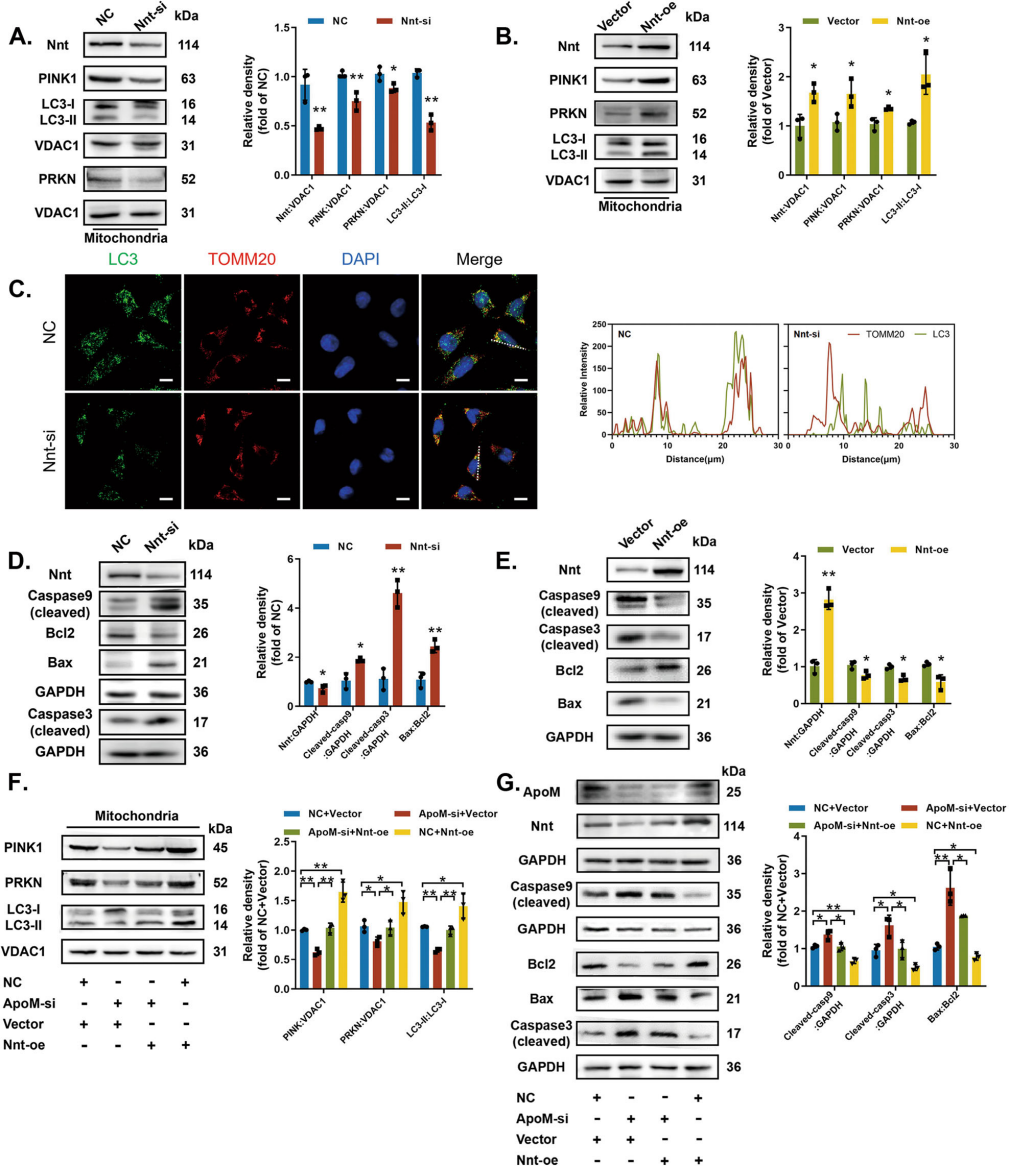
ApoM maintains cell homeostasis via Nnt. **A**, **C**, **D** C17.2 cells were transfected with Ncontrol (NC) or siRNA targeting Nnt (Nnt-si). **B**, **E** C17.2 cells were transfected with empty pcDNA vector (Vector) or WT Nnt cDNA (Nnt-oe). **A**, **B** PINK1, PRKN and LC3-II/LC3-I protein levels in mitochondria were measured by western blotting (*n* = 3 from independent experiments). **C** C17.2 cells were subjected to double IF staining with LC3-labelled autophagosomes (green) and TOMM20-labelled mitochondria (red). Nuclei were counterstained with DAP. Scale bar = 10 µm. **D**, **E** Nnt, Bax/Bcl-2, cleaved Caspase 9, and cleaved Caspase 3 protein levels were analysed by western blotting (*n* = 3 independent experiments). **F** PINK1, PRKN and LC3-II/LC3-I protein levels in mitochondria were analysed by western blotting under different transfection conditions in C17.2 cells (*n* = 3 from independent experiments). **G** Cleaved Caspase 9, cleaved Caspase 3, and Bax/Bcl-2 protein levels were analysed by western blotting under different transfection conditions in C17.2 cells (*n* = 3 from independent experiments). GAPDH or VDAC1 was used as a loading control. Data are expressed as the mean ± SD. Statistical comparisons were performed using Student’s *t* tests in (**A**, **B**, **D** and **E**); one-way ANOVA in (**F** and **G**). *P < 0.05; **P < 0.01; “ns” represents no significance vs. the indicated group.

After isolating mitochondrial proteins, we observed that silencing Nnt downregulated mitophagy activation markers (Fig. 4A), while overexpressing Nnt increased them (Fig. 4B). Silencing Nnt also led to low colocalization between LC3 and TOMM20 (Fig. 4C), whereas Nnt overexpression led to high colocalization (Fig. S2).

Subsequently, we observed that silencing Nnt increased the expression of apoptosis-related proteins (Fig. 4D); however, Nnt overexpression decreased them (Fig. 4E). These results suggest that Nnt maintains homeostasis between mitophagy and apoptosis.

Results from rescue experiments demonstrated that Nnt overexpression reversed ApoM-siRNA-induced downregulation of mitophagy activation markers in the mitochondria and upregulation of apoptosis-related proteins in the whole cells (Fig. 4F, G). Hence, ApoM appears to act on Nnt when disrupting the homeostasis between mitophagy and apoptosis.

### ApoM acts as a scaffold protein to recruit Elavl2 and maintain *Nnt* mRNA stability

**Fig. 5:**
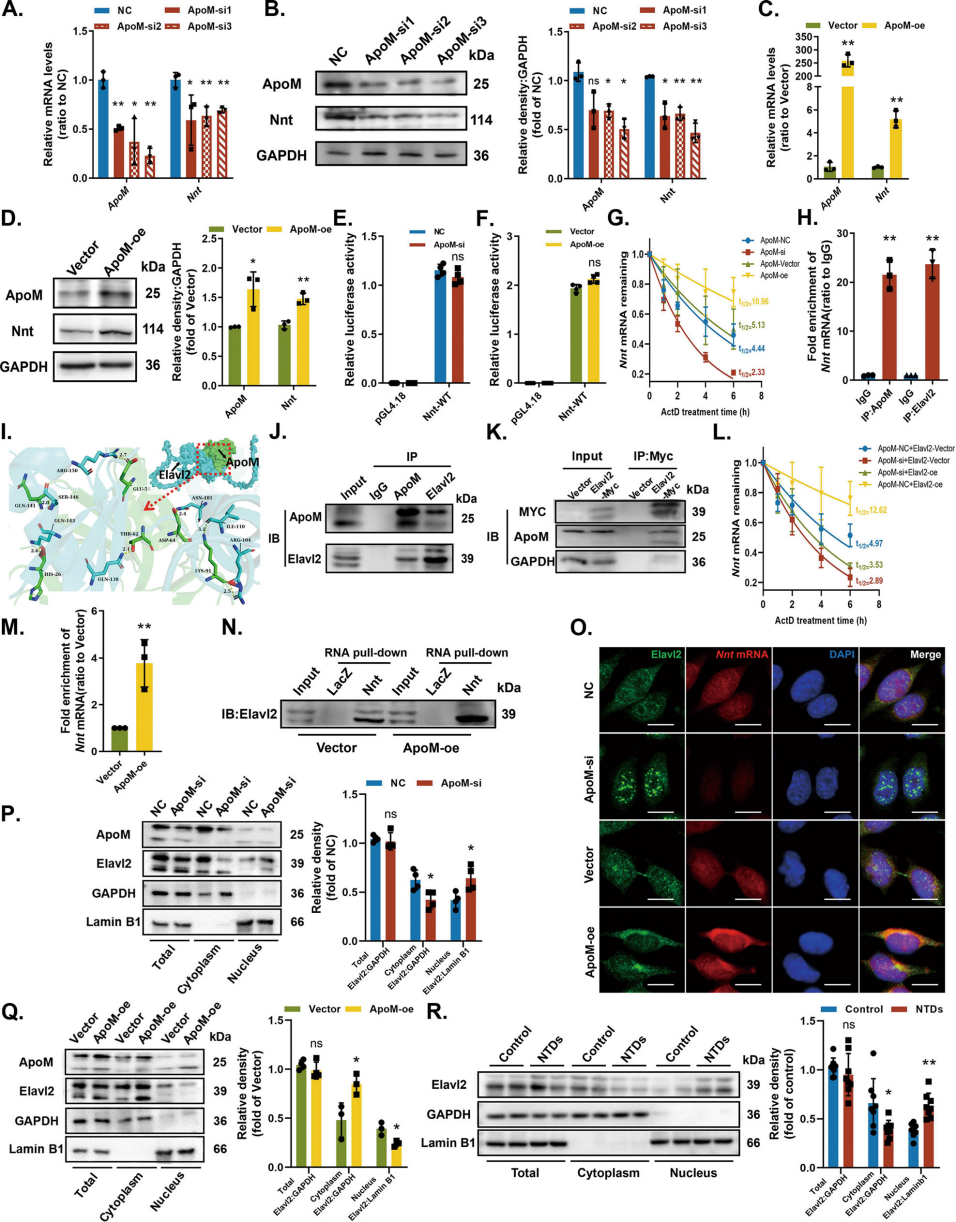
ApoM acts as a scaffold protein to recruit Elavl2 to stabilize *Nnt* mRNA. **A**, **B** C17.2 cells were transfected with Ncontrol (NC) or siRNA targeting ApoM (ApoM-si). **A** *ApoM* and *Nnt* mRNA levels were analysed by qRT-PCR (*n* = 3 from independent experiments). **B** ApoM and Nnt protein expressions were analysed by western blotting (*n* = 3 from independent experiments). **C**, **D** C17.2 cells were transfected with empty pcDNA vector (Vector) or WT ApoM cDNA (ApoM-oe). **C** *ApoM* and *Nnt* mRNA levels were analysed by qRT-PCR (*n* = 3 from independent experiments). **D** ApoM and Nnt protein expressions were analysed by western blotting (*n* = 5 from independent experiments). **E** Luciferase activity of the firefly luciferase construct without (pGL4.18) or with a mouse Nnt promoter (Nnt-WT) in C17.2 cells transfected with NC or ApoM-si (*n* = 4 from independent experiments). **F** Luciferase activity of the firefly luciferase construct without (pGL4.18) or with a mouse Nnt promoter (Nnt-WT) in C17.2 cells transfected with Vector or ApoM-oe (*n* = 4 from independent experiments). **G** C17.2 cells were incubated with Act D for indicated time periods to determine the stability of *Nnt* mRNA by qRT-PCR under different transfection conditions (*n* = 4 from independent experiments). **H** RIP assay determined the enrichment of *Nnt* mRNA by ApoM or Elavl2 in C17.2 cells (*n* = 3 from independent experiments). IgG was used as a negative control. **I** Simulation of protein docking between ApoM and Elavl2. **J** The endogenous interactions between ApoM and Elavl2 were examined by Co-IP in C17.2 cells. IgG was used as a negative control. **K** C17.2 cells were transfected with Vector or WT Elavl2-Myc cDNA (Elavl2-Myc), and exogenous interactions between ApoM and Elavl2 were detected by Co-IP. **L** C17.2 cells were incubated with Act D to determine the stability of *Nnt* mRNA by qRT-PCR under different transfection conditions (*n* = 4 from independent experiments). **M**, **N** C17.2 cells were transfected with Vector or ApoM-oe. **M** RIP assay determined the enrichment of *Nnt* mRNA by Elavl2 (*n* = 3 from independent experiments). **N** RNA pull-down analysis determined the enrichment of Elavl2 by biotin-labelled Nnt. Biotin-labelled LacZ was used as a negative control. **O**–**Q** C17.2 cells were transfected with NC, ApoM-si, Vector and ApoM-oe respectively. **O** The colocalization of *Nnt* mRNA (red) and Elavl2 (green) was detected by FISH and IF staining. Nuclei were counterstained with DAPI. Scale bar = 10 µm. **P**, **Q** Elavl2 protein levels in total cells, cytoplasm and nucleus were measured by western blotting (*n* = 3 from independent experiments). **R** Elavl2 protein expressions in total cells, cytoplasm and nucleus in the Control and NTDs rat models were analysed by western blotting (*n* = 8 per group). GAPDH or Lamin B1 was used as a loading control. Data are expressed as the mean ± SD. Statistical comparisons were performed using one-way ANOVA in (**A**, **B**); Student’s *t* tests in (**C**–**F**, **H**, **M** and **P**–**R**);.nonlinear regression in (**G** and **L**). *P < 0.05; **P < 0.01; “ns” represents no significance vs. the indicated group.

We investigated ApoM modulation of Nnt expression. After silencing ApoM in C17.2 cells. ApoM and Nnt expression was downregulated (Fig. 5A, B). Overexpressing ApoM upregulated *Nnt* in addition to *ApoM* (Fig. 5C, D).

To examine whether ApoM-enhanced Nnt expression occurs via a transcriptional mechanism, we cloned the full-length promoter region of *Nnt* mRNA and inserted it into the pGL4.18 luciferase reporter construct. Neither silencing (Fig. 5E) nor overexpressing ApoM (Fig. 5F) altered promoter activity. We then examined whether ApoM post-transcriptionally regulates Nnt expression. RNA stability measurements indicated that ApoM siRNA severely degraded *Nnt* mRNA, but ApoM overexpression suppressed the degradation (Fig. 5G).

We then explored how ApoM maintains *Nnt* mRNA stability. We used the RBPDB database (http://rbpdb.ccbr.utoronto.ca/) to predict RNA-binding proteins (RBPs) that were associated with *Nnt* mRNA (Supplementary Table S2). A high predictive score and importance for *Nnt* mRNA stability led us to select Elavl2 for subsequent analyses. Both ApoM and Elavl2 efficiently capture *Nnt* mRNA (RIP assay, Fig. 5H). Protein docking simulation based on PDB database (http://www.rcsb.org/) and AlphaFold protein structure data (https://alphafold.ebi.ac.uk/) showed that ApoM and Elavl2 interact via hydrogen bonds at GLN141-SER146, HIS26-GLN143, GLU58-ARG150, THR62-GLN138, ASP64-ASN101 and LYS91-ILE110/ARG104 (Fig. 5I). Endogenous and exogenous Co-IP assays further confirmed protein-protein interaction between ApoM and Elavl2 (Fig. 5J, K).

RNA stability measurement (Fig. 5L) further clarified that of Elavl2 overexpression increases *Nnt* mRNA stability. However, Elavl2 overexpression did not restore *Nnt* mRNA stability lost from ApoM silencing. Thus, ApoM affect *Nnt* mRNA stability via altering binding ability instead of Elavl2 expression. RIP and RNA pull-down assay further indicated that ApoM overexpression enhanced the direct interaction between *Nnt* mRNA and Elavl2 (Fig. 5M, N).

We also explored the effects of ApoM on Elavl2 subcellular localization. Silencing ApoM upregulated nuclear Elavl2 and downregulated *Nnt* mRNA throughout the cell, while also weakening Elavl2 and *Nnt* mRNA colocalization in the cytoplasm, and the opposite occurred after ApoM overexpression (RNA-FISH and IF staining, Fig. 5O). Western blotting further confirmed that silencing ApoM reduced cytoplasmic Elavl2 abundance and increased nuclear Elavl2 abundance, without altering total Elavl2 expression (Fig. 5P). Again, ApoM overexpression resulted in the opposite patterns for Elavl2 abundance (Fig. 5Q).

Data from NTDs rat models revealed a similar changes of Elavl2 expressions in the total, nuclear, and cytoplasmic proteins (Fig. 5R). Hence, ApoM is likely a scaffold protein for Elavl2, recruiting the latter to the cytoplasm for *Nnt* mRNA stabilization.

### ApoM is transcriptionally activated by Zic3

**Fig. 6:**
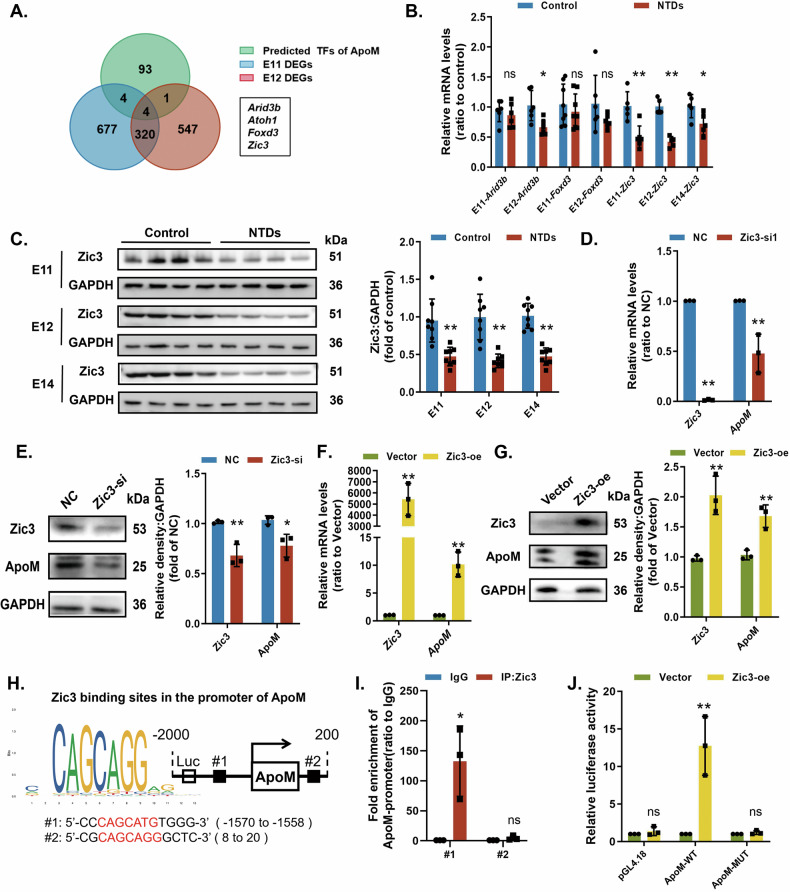
Zic3 positively regulates the transcription of ApoM. **A** Venn diagram shows the overlap between the transcription factors of ApoM predicted by the database and the DEGs at two time points of transcriptome sequencing of spinal cord from NTDs rat models. **B**, **C** Rat models were induced by a single intragastric administration of ATRA (NTDs) or olive oil (Control). The rat embryonic neural tube tissues were dissected on E11, E12 and E14. **B** *Arid3b, Foxd3* and *Zic3* mRNA expressions were assessed by qRT-PCR (*n* = 5–6 rats per group). **C** Zic3 protein expression was analysed by western blotting (*n* = 8 rats per group). **D**, **E** C17.2 cells were transfected with Ncontrol (NC) or siRNA targeting Zic3 (Zic3-si). **D** *Zic3* and *ApoM* mRNA levels were analysed by qRT-PCR (*n* = 3 from independent experiments). **E** Zic3 and ApoM protein expressions were analysed by western blotting (*n* = 3 from independent experiments). **F**, **G** C17.2 cells were transfected with an empty pcDNA vector (Vector) or WT Zic3 cDNA (Zic3-oe). **F** *Zic3* and *ApoM* mRNA levels were analysed by qRT-PCR (*n* = 3 from independent experiments). **G** Zic3 and ApoM protein expressions were analysed by western blotting (*n* = 3 from independent experiments). GAPDH was used as a loading control. **H** Diagram of the motifs recognised by Zic3 and the two predicted binding sites within the ApoM promoter, including 2000 bp upstream and 200 bp downstream. **I** ChIP analysis of Zic3 binding to the predicted sequence in the ApoM promoter in C17.2 cells (#1 and #2). IgG was used as a negative control (*n* = 3 from independent experiments). **J** Relative luciferase activity of the firefly luciferase construct without (pGL4.18) or with a promoter of the mouse ApoM gene (ApoM-WT) or Mut type in the #1 binding site of the ApoM promoter (ApoM-MUT) in C17.2 cells treated with Vector or Zic3-oe (*n* = 3 from independent experiments). Data are expressed as the mean ± SD. Statistical comparisons were performed using Student’s *t* tests. *P < 0.05; **P < 0.01; “ns” represents no significance vs. the indicated group.

*ApoM* transcription was downregulated in NTDs rat models, implying the involvement of transcription factors. We thus predicted candidate transcription factors of ApoM using the JASPER, PROMO and TF databases, matching them with DEGs [34] at E11 and E12 (Fig. 6A). Based on the regulatory roles of transcription factors and their trends in transcriptomics, Arid3b, Foxd3 and Zic3 were selected and validated in spinal cords. qRT-PCR showed that *Zic3* mRNA decreased in NTDs at different gestational ages, whereas *Arid3b* and *Foxd3* mRNA did not change significantly (Fig. 6B). Western blotting confirmed that Zic3 protein levels decreased in NTDs rats (Fig. 6C).

We then investigated Zic3 modulation of ApoM in vitro. ApoM expression decreased after silencing Zic3 (Fig. 6D, E), and increased with Zic3 overexpression (Fig. 6F, G). The JASPER database revealed that Zic3 recognize the conserved CAGCAGG motif, predicting two Zic3 binding sites in the ApoM promoter (Fig. 6H). The ChIP assay then showed that Zic3 specifically interacted with #1 binding sites (−1570 to −1558) of the ApoM promoter (Fig. 6I). The luciferase reporter assay demonstrated that Zic3 overexpression increased luciferase activity in the cells co-transfected with the full-length ApoM promoter region (ApoM-WT) but not in cells co-transfected with the Mut type of the #1 binding site (ApoM-Mut) (Fig. 6J). Taken together, these data indicate that Zic3 upregulates ApoM directly through its promoter.

### Zic3 maintains homeostasis via the ApoM-Nnt axis

**Fig. 7:**
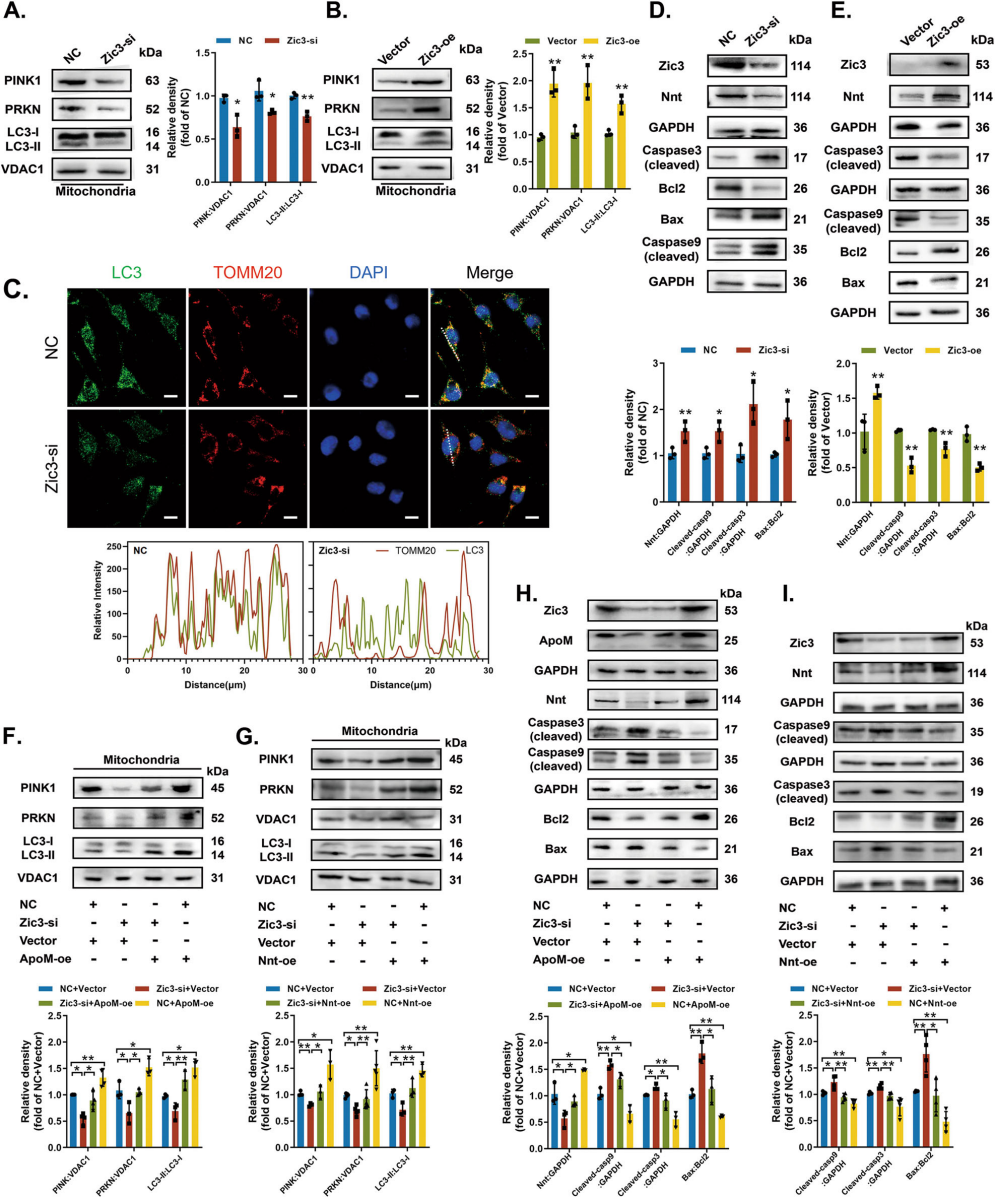
Zic3-ApoM-Nnt axis mediates cell homeostasis between mitophagy and apoptosis. **A**, **C**, **D** C17.2 cells were transfected with Ncontrol (NC) or siRNA targeting Zic3 (Zic3-si). **B**, **E** C17.2 cells were transfected with empty pcDNA vector (Vector) or WT Zic3 cDNA (Zic3-oe). **A**, **B** PINK1, PRKN and LC3-II/LC3-I protein levels in mitochondria were measured by western blotting (*n* = 3 from independent experiments). **C** C17.2 cells were double IF-stained with LC3-labelled autophagosomes (green) and TOMM20-labelled mitochondria (red). Nuclei were counterstained with DAPI. Scale bar = 10 µm. **D**, **E** Nnt, Bax/Bcl-2, cleaved Caspase 9, and cleaved Caspase 3 protein levels were analysed by western blotting (*n* = 3 independent experiments). **F**, **G** PINK1, PRKN and LC3-II/LC3-I protein levels in mitochondria were analysed by western blotting under different transfection conditions in C17.2 cells (*n* = 3 from independent experiments). **H**, **I** Nnt, cleaved Caspase 9, cleaved Caspase 3, and Bax/Bcl-2 protein levels were analysed by western blotting under different transfection conditions in C17.2 cells (*n* = 3 from independent experiments). GAPDH or VDAC1 was used as a loading control. Data are expressed as the mean ± SD. Statistical comparisons were performed using Student’s *t* tests in (**A**, **B**, **D** and **E**); one-way ANOVA in (**F** and **G**). *P < 0.05; **P < 0.01; “ns” represents no significance vs. the indicated group.

Mitophagy activation markers decreased after silencing Zic3 (Fig. 7A) but increased with Zic3 overexpression (Fig. 7B), indicating that Zic3 promotes PINK1-PRKN-mediated mitophagy. Silencing Zic3 also decreased colocalization between LC3 and TOMM20 (Fig. 7C), whereas Zic3 overexpression heightened colocalization (Fig. S3). Silencing Zic3 increased proapoptotic proteins (Fig. 7D), whereas Zic3 overexpression downregulated them (Fig. 7E). Therefore, Zic3 appears to inhibit cell apoptosis.

We performed rescue experiments to verify whether Zic3 affects mitophagy and apoptosis via the ApoM-Nnt axis. Overexpression of either ApoM or Nnt abrogated Zic3-silencing-related downregulation of mitophagy activation markers and upregulation of pro-apoptotic proteins (Fig. 7F–I). Therefore, Zic3 maintains cell homeostasis via the ApoM-Nnt axis.

### Zic3 or ApoM adenoviruses improve homeostasis between mitophagy and apoptosis

**Fig. 8:**
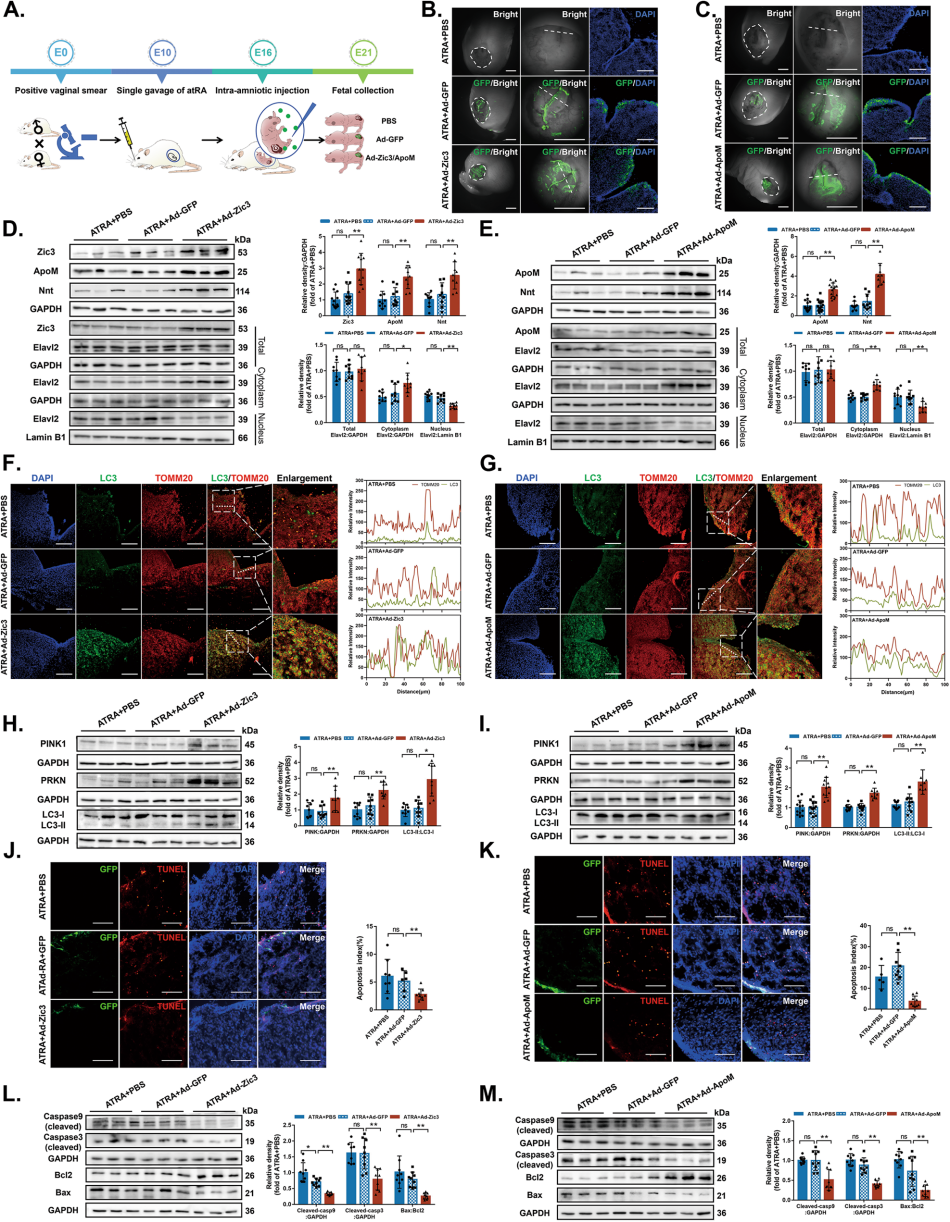
Intra-amniotic injections of Zic3 or ApoM adenoviruses exert therapeutic effects by improving cell homeostasis between mitophagy and apoptosis. **A** Schematic of intra-amniotic microinjections of Zic3 or ApoM adenoviruses. **B**, **C** Typical images of fluorescence stereomicroscope of embryos and fluorescence microscope of cryosections at E21 rat fetuses with NTDs after intra-amniotic injections of phosphate-buffered saline (PBS), GFP adenoviruses, Zic3 adenoviruses (**B**) or ApoM adenoviruses (**C**) The white dashed line circles the edge of the defective skin lesions (left column). Scale bar = 2 mm; the middle column is enlarged, and the white dashed line indicates the cryosections position; the right column shows the fluorescence of cryosections. Nuclei were counterstained with DAPI. Scale bar = 100 µm. **D**, **E** Zic3, ApoM, Nnt and Elavl2 protein expressions in different cell fractions in PBS-, Ad-GFP-, Ad-GFP-Zic3- (**D**) or Ad-GFP-ApoM-injected groups (**E**) were analysed by western blotting (*n* = 9 rats per group). **F**, **G** Cryosections of the spinal dorsal horn were double IF-stained with LC3-labelled autophagosomes (green) and TOMM20-labelled mitochondria (red) in the PBS-, Ad-GFP-, Ad-GFP-Zic3- (**F**) or Ad-GFP-ApoM-injected groups (**G**). Nuclei were counterstained with DAPI. Scale bar = 100 µm. **H**, **I** PINK1, PRKN and LC3-II/LC3-I protein levels in rat embryonic neural tube tissues were analysed by western blotting in the PBS-, Ad-GFP-, Ad-GFP-Zic3- (**H**) or Ad-GFP-ApoM-injected groups (**I**) (*n* = 9 rats per group). **J**, **K** Representative images and its quantification of TUNEL staining in the PBS-, Ad-GFP-, Ad-GFP-Zic3- (**J**) or Ad-GFP-ApoM-injected groups (**K**). Nuclei were counterstained with DAPI. Scale bar = 100 µm (*n* = 5–8 rats per group). **L**, **M** Cleaved Caspase 9, cleaved Caspase 3 and Bax/Bcl-2 protein levels in rat embryonic neural tube tissues were analysed by western blotting in the PBS-, Ad-GFP-, Ad-GFP-Zic3- (**L**) or Ad-GFP-ApoM-injected groups (**M**) (*n* = 9 rats per group). GAPDH was used as a loading control. Data are expressed as the mean ± SD. Statistical comparisons were performed using one-way ANOVA. *P < 0.05; **P < 0.01; “ns” represents no significance vs. the indicated group.

After establishing the Zic3-ApoM-Elavl2-Nnt axis, we selected Zic3 and ApoM as key therapeutic targets for NTDs. To investigate the role of Zic3 and ApoM in vivo, we injected adenoviral vectors expressing either Zic3 (Ad-GFP-Zic3) or ApoM (Ad-GFP-ApoM) into fetal rats with NTDs on E16 (Fig. 8A). We observed GFP fluorescence in the defective regions of NTDs whole fetuses and in fetal cryosections for the Ad-GFP-Zic3 (Fig. 8B) and Ad-GFP-ApoM groups (Fig. 8C) respectively, which indicated that adenoviruses had been successfully transfected.

After intra-amniotic gene therapy, western blotting revealed that Zic3, ApoM and Nnt levels in the fetal neural tube were significantly higher for the Ad-GFP-Zic3-injected group than for the PBS- or Ad-GFP-injected groups. We also isolated nuclear and cytoplasmic proteins from the fetal spinal cord. Total Elavl2 abundance did not change significantly after gene therapy, while cytoplasmic Elavl2 abundance increased (Fig. 8D). Similarly, ApoM and Nnt rose significantly in the Ad-ApoM-injected group, as did cytoplasmic Elavl2 abundance (Fig. 8E).

Stained cryosections for LC3 and TOMM20 revealed greater colocalization in the Ad-GFP-Zic3- (Fig. 8F) and Ad-GFP-ApoM-injected groups (Fig. 8G) than in the PBS- or Ad-GFP-injected groups. Western blotting further showed that mitophagy activation markers in the neural tube were increased in the Ad-GFP-Zic3- (Fig. 8H) and Ad-GFP-ApoM-injected groups (Fig. 8I).

After staining cryosections with TUNEL, we observed a reduction in apoptotic cells from the Ad-GFP-Zic3- (Fig. 8J) and Ad-GFP-ApoM-injected groups (Fig. 8K). Western blotting further indicated that pro-apoptotic proteins decreased in the Ad-GFP-Zic3- (Fig. 8L) and Ad-GFP-ApoM-injected groups (Fig. 8M). Together, these findings imply that intra-amniotic injections of Zic3 or ApoM adenoviruses improve cell homeostasis between mitophagy and apoptosis in fetal rats with NTDs.

## Discussion

Our study offered empirical evidence of ApoM downregulation in the spinal cords of NTDs rat models and NTDs human fetuses. In addition to demonstrating the essential role of ApoM in neural tube closure, we clarified its mechanism of action: ApoM alters Elavl2 subcellular localization to maintain the stability of *Nnt* mRNA. Our findings highlighted a new function of ApoM in subcellular protein localization and post-transcriptional regulation of genes. We also noted that Zic3 regulates ApoM, allowing us to establish a novel Zic3-ApoM-Elavl2-Nnt axis. In vitro functional experiments and in vivo intra-amniotic gene therapy emphasized the critical role of the Zic3-ApoM-Elavl2-Nnt axis in maintaining cell homeostasis between mitophagy and apoptosis during neural tube development. Our results provide a mechanistic basis for embryonic neural development and provides experimental evidence for the potential therapeutic targets of NTDs.

ApoM is centrally involved in regulating cholesterol metabolism and turnover, while also being implicated in the onset of Alzheimer’s disease [36, 37]. Our study confirmed that ApoM is downregulated in both animal models and patients with NTDs. During human embryogenesis, ApoM is mainly expressed in the liver and kidneys, with trace amounts also present in the small intestine, stomach and skeletal muscle [38]. Notably, the study also found that ApoM expression was low at E7.5 and significantly elevated at E9.5, suggesting that ApoM plays an important role in this developmental stage, which is also a critical period for neural tube formation and closure. During this critical period, the lower expression of ApoM in embryonic spinal cord tissue may be the cause of NTD occurrence.

Accumulating evidence suggests that ApoM improves mitochondrial function, exerts anti-apoptosis and antioxidative effects and participates in pathological processes (including diabetic nephropathy and atherosclerosis) [39, 40]. Our study supports ApoM involvement in mitochondrial function. ApoM deficiency induces an imbalance between mitophagy and apoptosis. Cellular homeostasis involving autophagy and apoptosis is critical for embryonic development [41, 42]. Previously we demonstrated that resveratrol reduces NTDs occurrence via ameliorating mitophagy damage [34]. Mitophagy selectively removes excess or damaged mitochondria to control mitochondrial quality [35]. PINK1-PRKN pathway is a classical mitophagy pathways. Under stress, PINK1 localizes to the outer mitochondrial membrane and recruits parkin. The outer membrane components are ubiquitinated by parkin and phosphorylated by PINK1, generating an autophagy signal that finally binds to LC3 to form autophagosomes. In the present study, we observed that ApoM deficiency reduced mitophagosomes count and inhibited PINK1-PRKN-mediated mitophagy. These changes negatively affect removal of damaged mitochondria and harmful components, disrupting cell homeostasis and ultimately triggering apoptosis.

The underlying mechanism of ApoM action in neural tube formation has largely been unexplored. In our study, we demonstrated that the ApoM-Nnt axis is heavily involved in neural tube closure. We successfully verified the downregulation of Nnt in NTD rat models after a screening process based on the top 10 DEGs in ApoM^(−/−)^ mouse [33] and a transcriptome analysis [34]. Nnt is an important mitochondrial enzyme involved in the tricarboxylic acid cycle [43], which drives processes such as ROS detoxification and reductive biosynthesis, and maintains mitochondrial function [44, 45]. We then investigated the effects of Nnt on cellular homeostasis. Nnt inhibits apoptosis by maintaining mitochondrial membrane potential. A lack of Nnt activity can impair peroxide metabolism in the mitochondria, leading to oxidative stress [46]. To date, only one study has reported the role of Nnt in tumor cell autophagy [47], and none have explored the relationship between Nnt and mitophagy. Our study confirmed that Nnt partially rescued the PINK1-PRKN-mediated decrease in mitophagy and the increase in apoptosis, clarifying the importance of ApoM-Nnt in regulating cellular homeostasis in NTDs.

Further research is needed to elucidate the molecular mechanisms underlying ApoM regulation of Nnt expression.ApoM binds to S1P, myristic acid, and other active substances and is mainly involved in lipid transport [23, 48, 49]. ApoM may have evolved additional functions apart from lipid transport. For example, ApoM appears to interact with the E2 protein and participate in the assembly/release step of hepatitis C virus life cycle. However, the domain (aa 76–105) required for the ApoM-E2 interaction did not overlap with any of ApoM’s three conserved domains, providing clues for the discovery of new functions of ApoM [30]. This novel domain thus provides clues for new functions. Our study demonstrated that ApoM affects *Nnt* mRNA stability and post-transcriptically regulates Nnt expression. AU-rich elements regulates mRNA stability at the post-transcriptional level, recruiting specific proteins to regulate their own degradation [50–52]. RBPs are key to gene regulatory processes, including RNA synthesis, alternative splicing, stability, modification, transport and translation [53, 54]. Here, we found that the RBP, Elavl2, maintains *Nnt* mRNA stability. Depending on the type of RNA-binding protein and target RNA, RNA decay can occur in the cytoplasm or nucleus [55]. Elavl2, a member of the Hu protein family, competes with AU-rich elements-targeted proteins in the cytoplasm to suppress transcriptional decay and maintain mRNA stability [56]. Our study revealed that ApoM interacts with Elavl2 to localize the latter in the cytoplasm. This interaction allows Elavl2 to bind and stabilize *Nnt* mRNA, thus regulating gene expression at the post-transcriptional level. In summary, our study demonstrates that ApoM is involved in subcellular protein localization and the regulation of gene expression, in addition to its traditional function as a transporter.

Abnormal transcription factors are a major genetic causes of NTDs [57]. Herein, we identified Zic3 as a potential transcription factor of ApoM. Zic3 is a member of the Zic family and associated with birth defects [58]. Zic3-knockout mice develope phenotypes of anencephaly, spina bifida and curly tails [58, 59]. Mutations in human ZIC3 also cause NTDs [60]. This study is the first to clarify the molecular mechanisms underlying Zic3 participation in NTDs. As expected, Zic3 bound directly to the ApoM promotor to trigger ApoM transcription. Additionally, Zic3 promoted PINK1-PRKN-mediated mitophagy and suppressed apoptosis via ApoM. These results are consistent with prior research demonstrating that Zic3 is related to oxidative stresss-induced apoptosis [61].

Intra-amniotic injections of adenoviruses can achieve sustained gene expression in the neural tube during the early stages of fetal development, reduce damage to mother and fetus, and improve the efficacy of intrauterine transgene therapy [62, 63]. Our previous studies used intra-amniotic injections targeting CRMP4 and BDNF to repair various tissue defects in NTD rats [31, 64–66]. We also used this approach to deliver miR-322 mimics targeting NOX4 in NTDs mice to alleviate apoptosis [67], suggesting that intra-amniotic injection is an effective gene therapy approach for NTDs. In our study, intra-amniotic delivery of adenoviral recombinant Zic3 or ApoM had therapeutic effects in NTDs rat models. The same method may be a promising strategy for the treatment of NTDs.

This study has several limitations. First, we only investigated apoptosis, despite the existence of a wide range of cell death types [68]. ApoM appears to limit pyroptosis and improve inflammation in human umbilical vein endothelial cells during atherosclerosis [69]. Additional studies have revealed the potential value of ApoM in impeding liver-cancer cell metastasis through facilitating ferroptosis [70]. Nnt is also associated with ferroptosis, maintaining iron-sulfur clusters that influence tumor progression [71]. In the further study we needs to more broadly validate the effects of the Zic3-ApoM-Nnt axis on other forms of cell death. Second, we did not validate the effects of the Zic3-ApoM-Nnt axis in other cell models. Third, we only used human fetuses with spina bifida at 24–33 weeks of pregnancy to verify ApoM expression. In the future, human NTD specimens at 3–5 weeks, which is the critical period of neural tube formation, can be used to confirm the expression of key factors and better understand the molecular mechanisms underlying neural tube closure.

This study highlights the fact that an abnormal Zic3-ApoM-Elavl2-Nnt axis may lead to NTDs formation via inhibiting mitophagy and triggering apoptosis. In addition to its classic apolipoprotein function, we identified a new function of ApoM that affects Elavl2 localization and regulates *Nnt* mRNA stability at the post-transcriptional level. The therapeutic effects of Zic3 and ApoM on NTDs were confirmed in vivo. These findings provide insights into NTDs pathogenesis and potential therapeutic targets.

## Supplementary information

Supplementary Table 1

Supplementary Table 2

Supplementary Table 3

Figure S1

Figure S2

Figure S3

Supplementary legend

Supplemental Material – Original Blots

## Data Availability

All data generated or analysed during this study are included in this published article and its supplementary files.
