# Supplementary material for: ApoM maintains cellular homeostasis between mitophagy and apoptosis by affecting the stability of Nnt mRNA through the Zic3-ApoM-Elavl2-Nnt axis during neural tube closure
Source: Cell Death Dis. 2025 Jan 19;16(1):29. doi: 10.1038/s41419-025-07343-3 (PMC11742887; doi:10.1038/s41419-025-07343-3)
Supplement: Supplementary file 1 — Supplementary Table 1 [file 41419_2025_7343_MOESM1_ESM.docx]

Supplementary Table 1. Summary of clinical features and demographic information of samples in this study

| **Group** | **Sample Lable** | **Types of deformity** | **Sex** | **Gestational weeks** |
| --- | --- | --- | --- | --- |
| Control Group | S189 | Inevitable abortion | F | 26+5 |
|  | S158 | Unplanned pregnancy | M | 24+1 |
|  | S108 | Inevitable abortion | F | 25+6 |
|  | 807416 | Inevitable abortion | F | 28+6 |
|  | 707666 | Mild pericardial effusion | F | 30+3 |
|  | S90 | Unplanned pregnancy | M | 34 |
| NTDs Group | ES4 | Spina bifida | F | 27+6 |
|  | ES9 | Spina bifida | M | 24 |
|  | ES1 | Meningomyelocele | F | 26 |
|  | ES3 | Spina bifida | F | 29+3 |
|  | ES6 | Spina bifida | F | 30 |
|  | ES7 | Spina bifida | M | 33 |
