# Supplementary material for: ApoM maintains cellular homeostasis between mitophagy and apoptosis by affecting the stability of Nnt mRNA through the Zic3-ApoM-Elavl2-Nnt axis during neural tube closure
Source: Cell Death Dis. 2025 Jan 19;16(1):29. doi: 10.1038/s41419-025-07343-3 (PMC11742887; doi:10.1038/s41419-025-07343-3)
Supplement: Supplementary file 2 — Supplementary Table 2 [file 41419_2025_7343_MOESM2_ESM.docx]

| RBP Name | Score | Start | End | Matching sequence |
| --- | --- | --- | --- | --- |
| SFRS2 | 15.12716346 | 3720 | 3729 | UGUUCGAGAU |
| [ELAVL2](http://rbpdb.ccbr.utoronto.ca/proteins.php?PME_sys_operation=PME_op_View&PME_sys_rec=1344) | 13.36588376 | 1141 | 1149 | UUUUAUUUA |
| [ZFP36](http://rbpdb.ccbr.utoronto.ca/proteins.php?PME_sys_operation=PME_op_View&PME_sys_rec=1405) | 12.73940513 | 5331 | 5341 | AAAAAAAAAAG |
| [A2BP1](http://rbpdb.ccbr.utoronto.ca/proteins.php?PME_sys_operation=PME_op_View&PME_sys_rec=1276) | 11.070935 | 2313 | 2318 | UGCAUG |
| [ELAVL2](http://rbpdb.ccbr.utoronto.ca/proteins.php?PME_sys_operation=PME_op_View&PME_sys_rec=1344) | 10.9852169 | 3963 | 3971 | UUCUAUUUU |
| [SFRS2](http://rbpdb.ccbr.utoronto.ca/proteins.php?PME_sys_operation=PME_op_View&PME_sys_rec=1537) | 10.97654042 | 4798 | 4806 | AGGAGAGGA |
| [SFRS2](http://rbpdb.ccbr.utoronto.ca/proteins.php?PME_sys_operation=PME_op_View&PME_sys_rec=1537) | 10.59224079 | 3721 | 3729 | GUUCGAGAU |
| [ELAVL2](http://rbpdb.ccbr.utoronto.ca/proteins.php?PME_sys_operation=PME_op_View&PME_sys_rec=1344) | 10.44817017 | 1377 | 1385 | UUUCACUUU |
| [ZRANB2](http://rbpdb.ccbr.utoronto.ca/proteins.php?PME_sys_operation=PME_op_View&PME_sys_rec=2917) | 10.3039431 | 1124 | 1129 | AGGUAA |
| [SNRPA](http://rbpdb.ccbr.utoronto.ca/proteins.php?PME_sys_operation=PME_op_View&PME_sys_rec=1274) | 10.1470304 | 349 | 355 | AGGAGAU |

Supplementary Table 2. Candidate RNA-binding protein of *Nnt* mRNA predicted by RBPDB database
