# Supplementary material for: ApoM maintains cellular homeostasis between mitophagy and apoptosis by affecting the stability of Nnt mRNA through the Zic3-ApoM-Elavl2-Nnt axis during neural tube closure
Source: Cell Death Dis. 2025 Jan 19;16(1):29. doi: 10.1038/s41419-025-07343-3 (PMC11742887; doi:10.1038/s41419-025-07343-3)
Supplement: Supplementary file 3 — Supplementary Table 3 [file 41419_2025_7343_MOESM3_ESM.docx]

Supplementary Table 3. The sequences of primers used in qRT-PCR

| Genes | Species | Primers | Sequence (5’-3’) |
| --- | --- | --- | --- |
| GAPDH | Rat | Forward | TGCCGCCTGGAGAAACCTGC |
|  |  | Reverse | AGCAATGCCAGCCCCAGCAT |
|  |  |  |  |
| GAPDH | Mouse | Forward | GTTGTCTCCTGCGACTTCA |
|  |  | Reverse | TGGTCCAGGGTTTCTTACTCC |
|  |  |  |  |
| ApoM | Rat | Forward | GCTGGGAATGGACGACAAAGAGAC |
|  |  | Reverse | CCGGCGGCCATGTTGAAGAC |
|  |  |  |  |
| ApoM | Mouse | Forward | AAGAGTTGGCAACTTTTGATCC |
|  |  | Reverse | GTTCCATGTTTCCTTTCCCTTC |
|  |  |  |  |
| Nnt | Rat | Forward | GTTGCCTTGTCTCCTGCTGGTG |
|  |  | Reverse | GCTTCGCCTGCTCCTGATTCC |
|  |  |  |  |
| Nnt | Mouse | Forward | AGATGTCTGGAGCAATGGCAATGG |
|  |  | Reverse | CTGAGGCAAGTCGGAAATCTGGATC |
|  |  |  |  |
| Onecut1 | Rat | Forward | GGTCTGGGCAGCATTCACAACTC |
|  |  | Reverse | GCAGGGTGGTGGGCTTCAAAG |
|  |  |  |  |
| Zic3 | Rat | Forward | CTGCCGCCTTCAAGCTGAGC |
|  |  | Reverse | CGTGATGGTGGTGGTGGTGATG |
|  |  |  |  |
| Zic3 | Mouse | Forward | GCAGGCATGGGATTGAATC |
|  |  | Reverse | ATTGGCATAACCTGAACCCTG |
|  |  |  |  |
| Elavl2 | Mouse | Forward | CACAGAGGACAGCAAGACCAACC |
|  |  | Reverse | TTACAGGACTCTATCTCGCCAATGC |
|  |  |  |  |
| ChIP Site 1#  cccagcatgtggg | Mouse | Forward | CTGCCAAGTACCCTCACTG |
|  |  | Reverse | CTCAGAAATCCGCCTGCC |
|  |  |  |  |
| ChIP Site 2# cgcagcagggctc | Mouse | Forward | CCAGTGAATGAATGAATGAAAGACC |
|  |  | Reverse | CCAGTGACTCTGAGGCCTTAG |
