# Supplementary figures and images for: ApoM maintains cellular homeostasis between mitophagy and apoptosis by affecting the stability of Nnt mRNA through the Zic3-ApoM-Elavl2-Nnt axis during neural tube closure
Source: Cell Death Dis. 2025 Jan 19;16(1):29. doi: 10.1038/s41419-025-07343-3 (PMC11742887; doi:10.1038/s41419-025-07343-3)

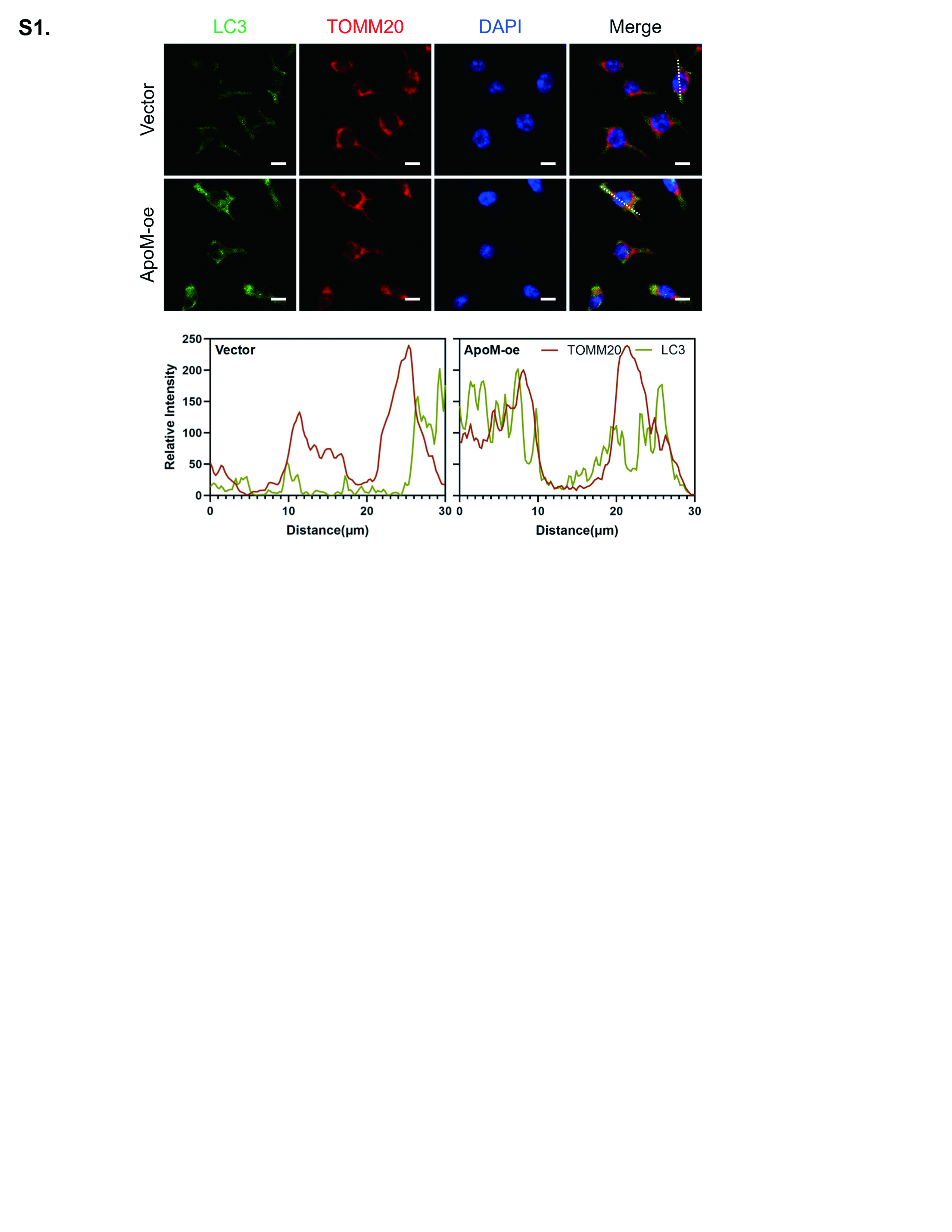

Supplement: Supplementary file 4 — Figure S1 [file 41419_2025_7343_MOESM4_ESM.tif]

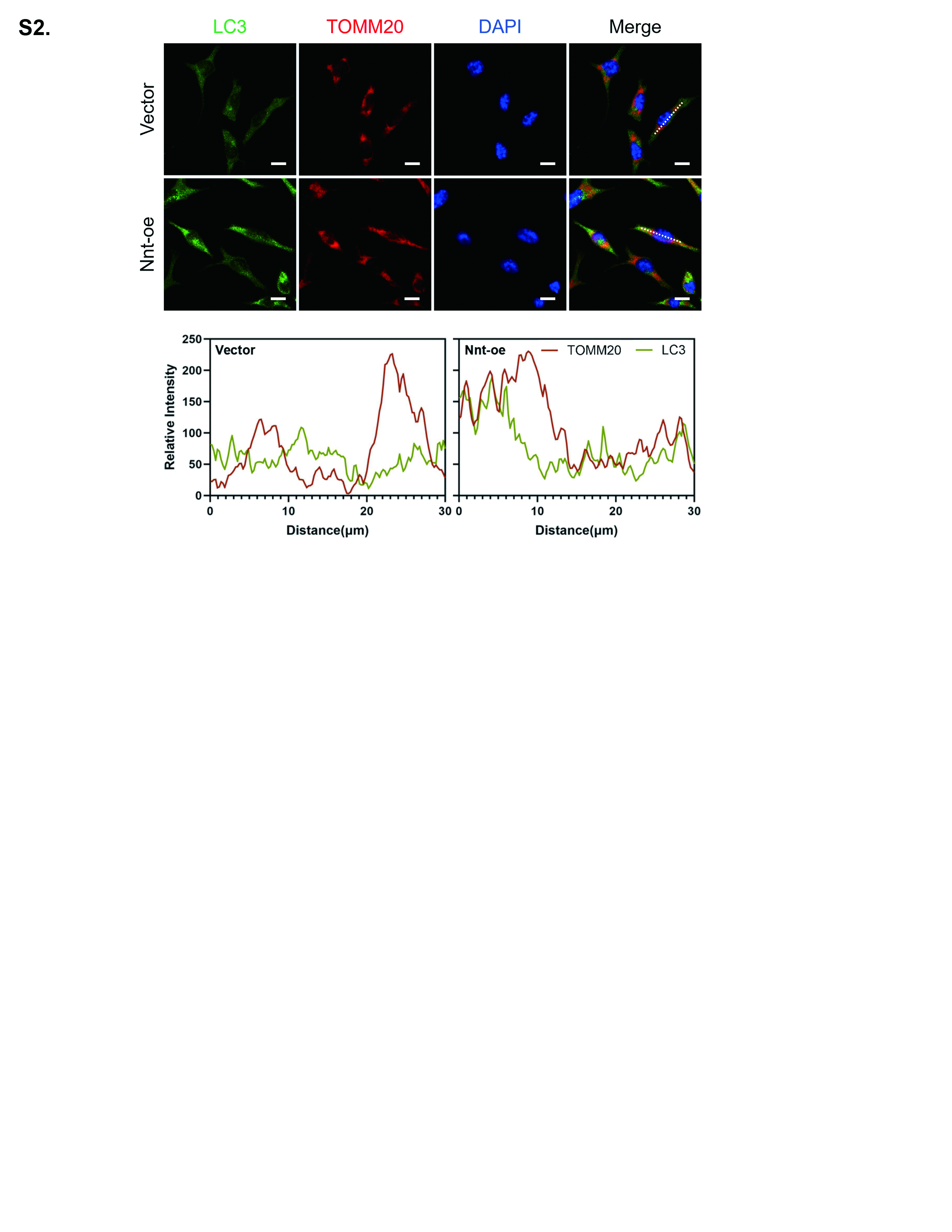

Supplement: Supplementary file 5 — Figure S2 [file 41419_2025_7343_MOESM5_ESM.tif]

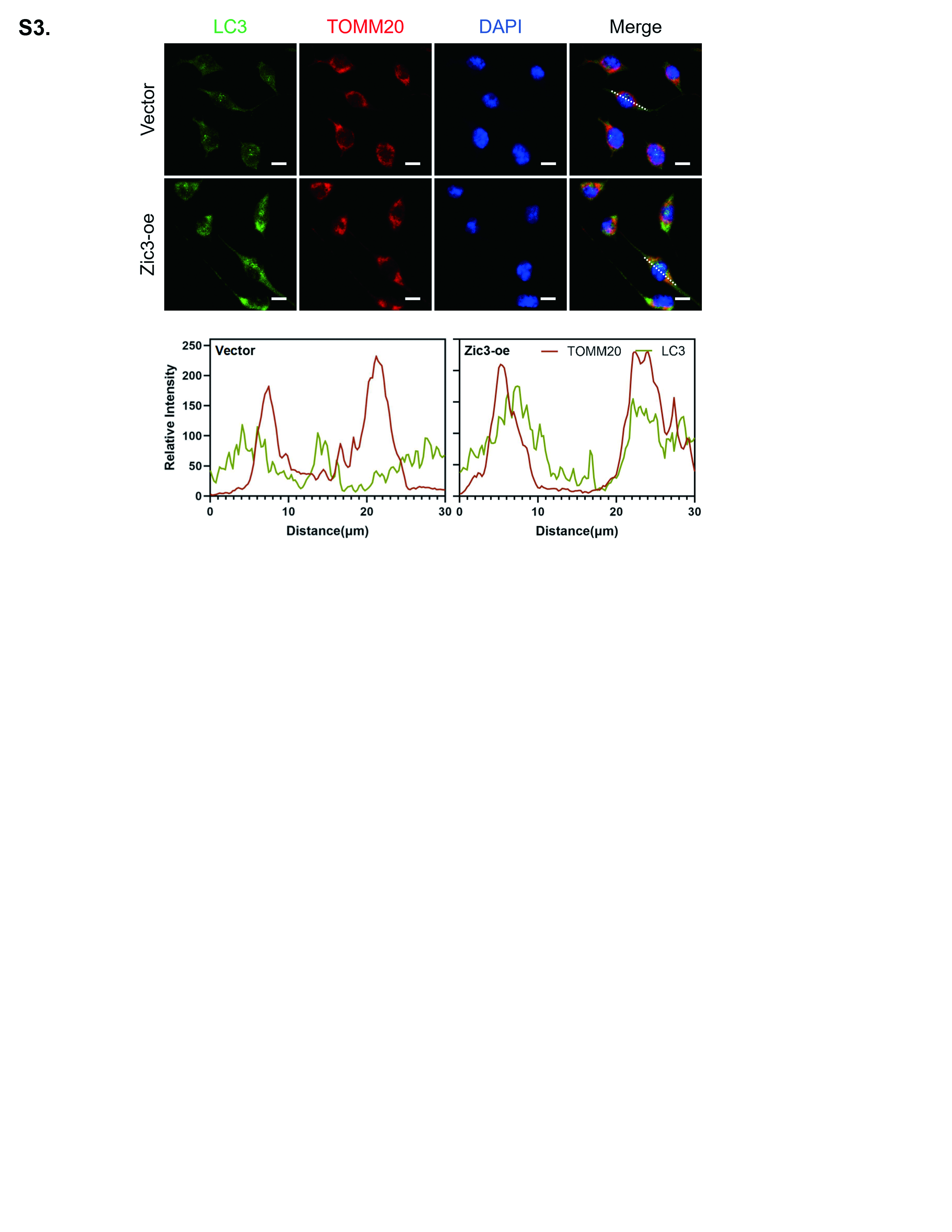

Supplement: Supplementary file 6 — Figure S3 [file 41419_2025_7343_MOESM6_ESM.tif]
