# Supplementary material for: ApoM maintains cellular homeostasis between mitophagy and apoptosis by affecting the stability of Nnt mRNA through the Zic3-ApoM-Elavl2-Nnt axis during neural tube closure
Source: Cell Death Dis. 2025 Jan 19;16(1):29. doi: 10.1038/s41419-025-07343-3 (PMC11742887; doi:10.1038/s41419-025-07343-3)
Supplement: Supplementary file 7 — Supplementary legend [file 41419_2025_7343_MOESM7_ESM.docx]

**Fig. S1. Overexpression of ApoM promotes co-localization of LC3 and TOMM20.**

C17.2 cells transfected with empty pcDNA vector (Vector) or WT ApoM cDNA (ApoM-oe) were double IF-stained with LC3-labelled autophagosomes (green) and TOMM20-labelled mitochondria (red). Nuclei were counterstained with DAPI. Scale bar = 10 µm.

**Fig. S2. Overexpression of Nnt promotes co-localization of LC3 and TOMM20.**

C17.2 cells transfected with empty pcDNA vector (Vector) or WT Nnt cDNA (Nnt-oe) were double IF-stained with LC3-labelled autophagosomes (green) and TOMM20-labelled mitochondria (red). Nuclei were counterstained with DAPI. Scale bar = 10 µm.

**Fig. S3. Overexpression of Zic3 promotes co-localization of LC3 and TOMM20.**

C17.2 cells transfected with empty pcDNA vector (Vector) or WT Zic3 cDNA (Zic3-oe) were double IF-stained with LC3-labelled autophagosomes (green) and TOMM20-labelled mitochondria (red). Nuclei were counterstained with DAPI. Scale bar = 10 µm.
