# Supplementary material for: ApoM maintains cellular homeostasis between mitophagy and apoptosis by affecting the stability of Nnt mRNA through the Zic3-ApoM-Elavl2-Nnt axis during neural tube closure
Source: Cell Death Dis. 2025 Jan 19;16(1):29. doi: 10.1038/s41419-025-07343-3 (PMC11742887; doi:10.1038/s41419-025-07343-3)
Supplement: Supplementary file 8 — Supplemental Material – Original Blots [file 41419_2025_7343_MOESM8_ESM.pdf]

# ApoM maintains cellular homeostasis between mitophagy and apoptosis by affecting the stability of *Nnt* mRNA through the Zic3-ApoM-Elavl2-Nnt axis during neural tube closure

Supplemental Material – Original Blots

Relevant areas for cropped blots in the figures are indicated by red boxes or arrows.

**Figure 1 B**

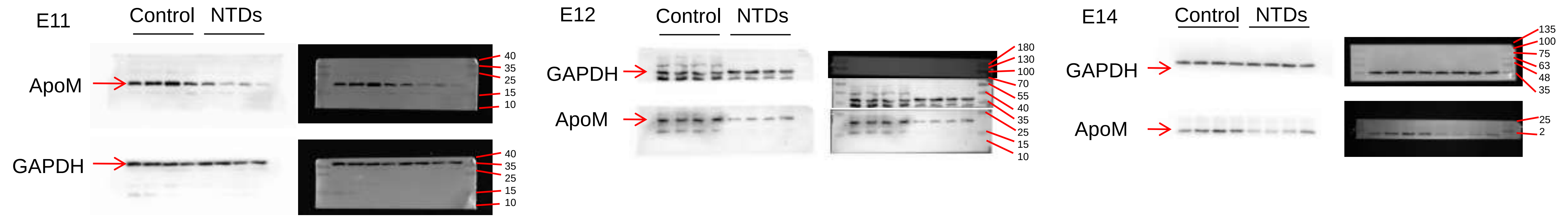


**Figure 1 C**

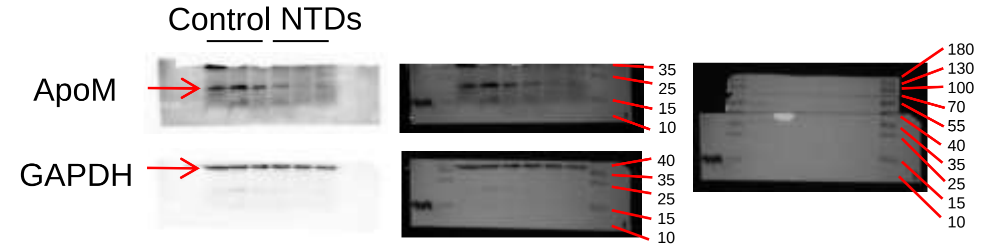


**Figure 1 F**

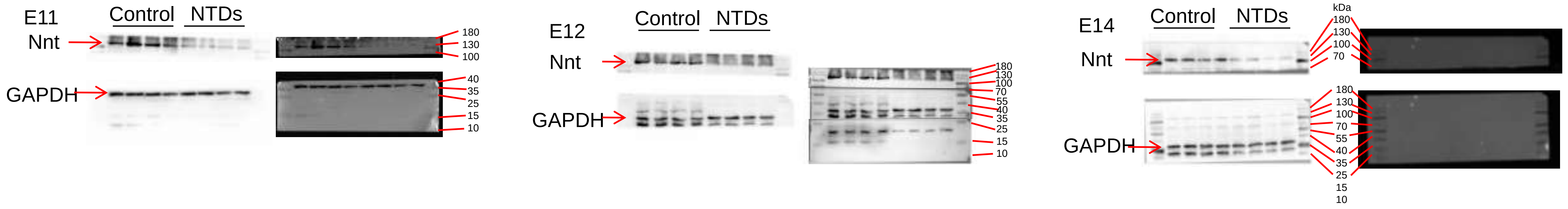

Figure 3 A

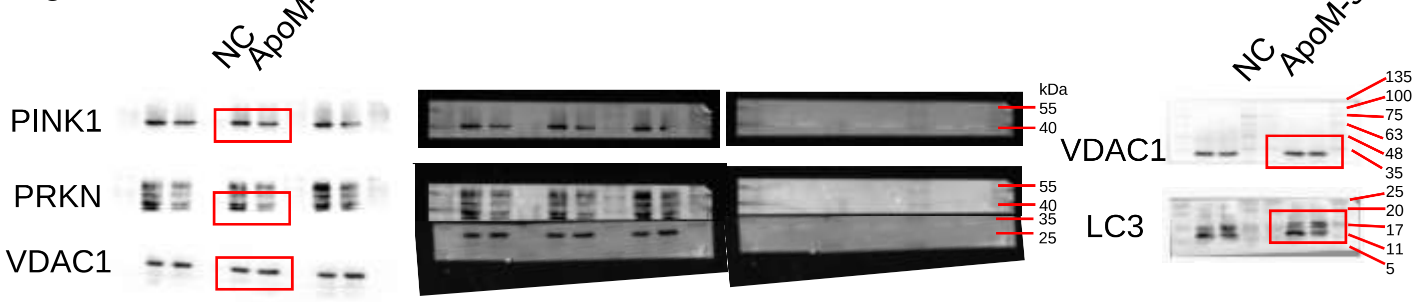


Figure 3 B

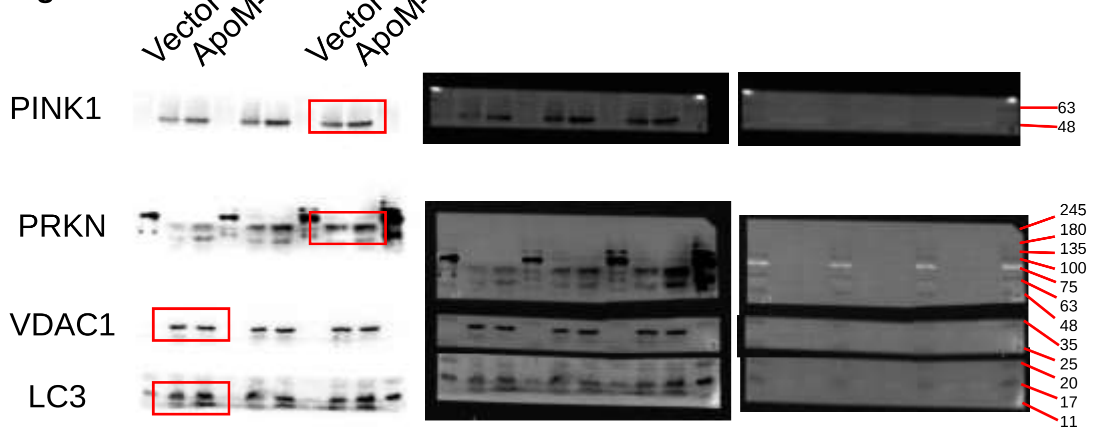


Figure 3 D

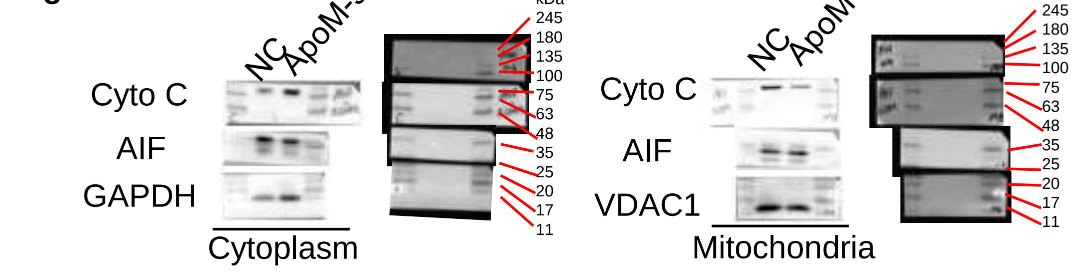


Figure 3 E

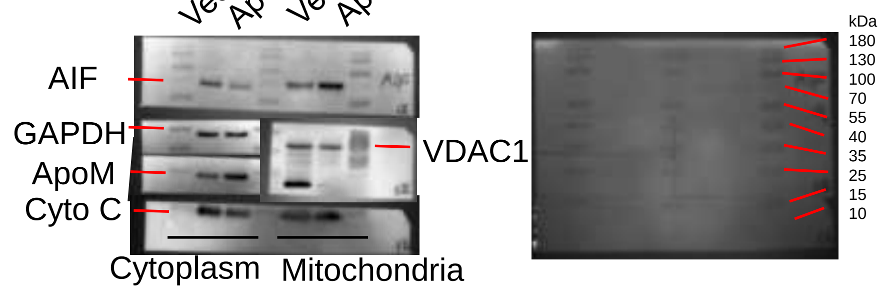

**Figure 3 F**

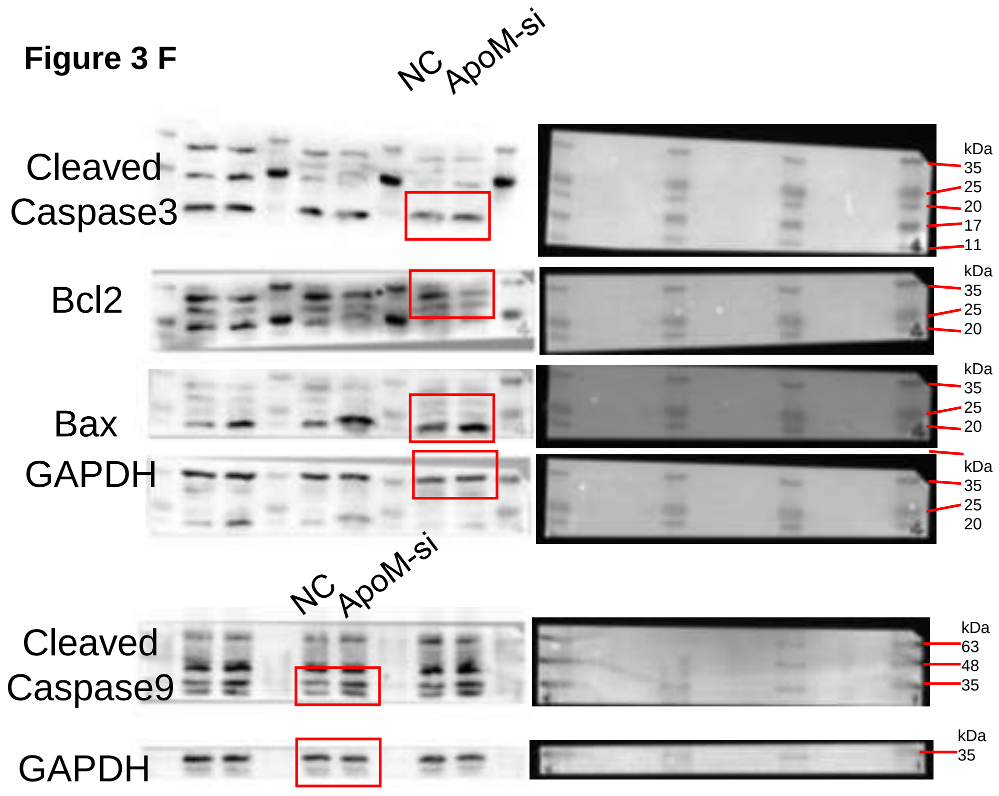


**Figure 3 G**

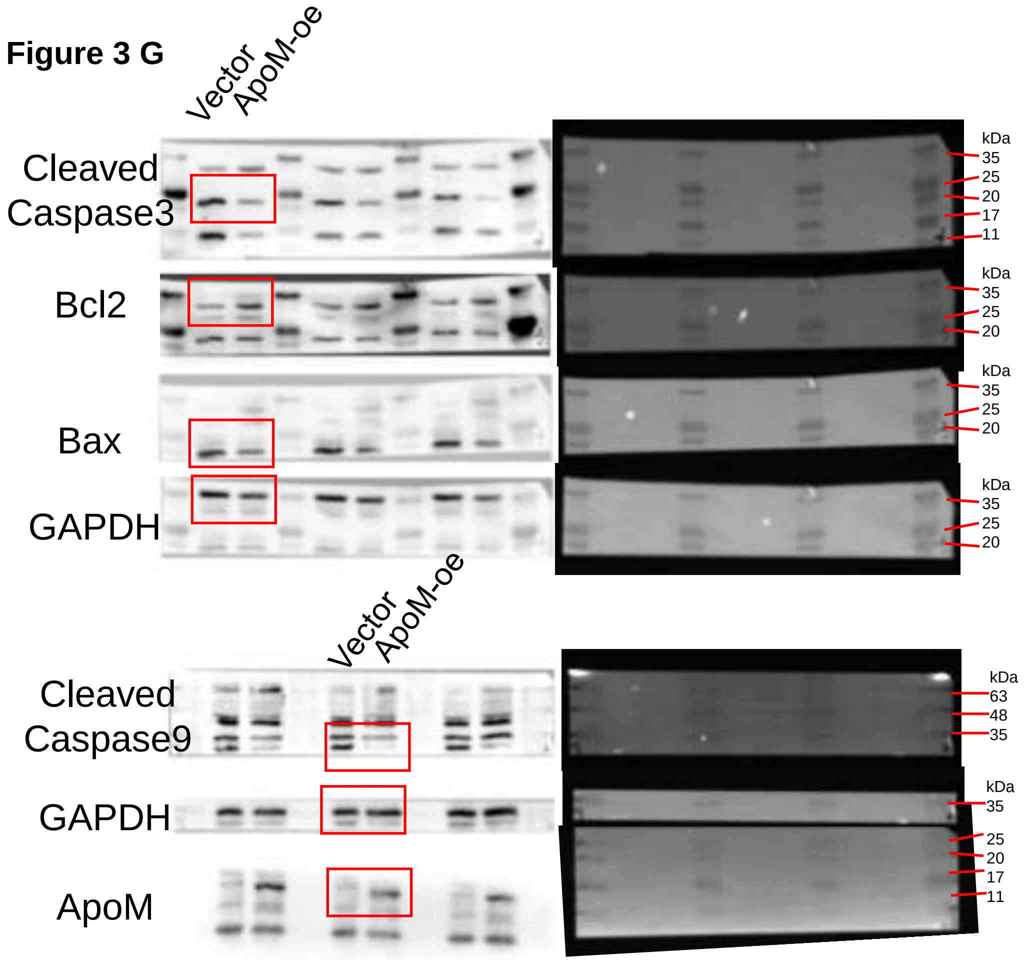

**Figure 3 H**

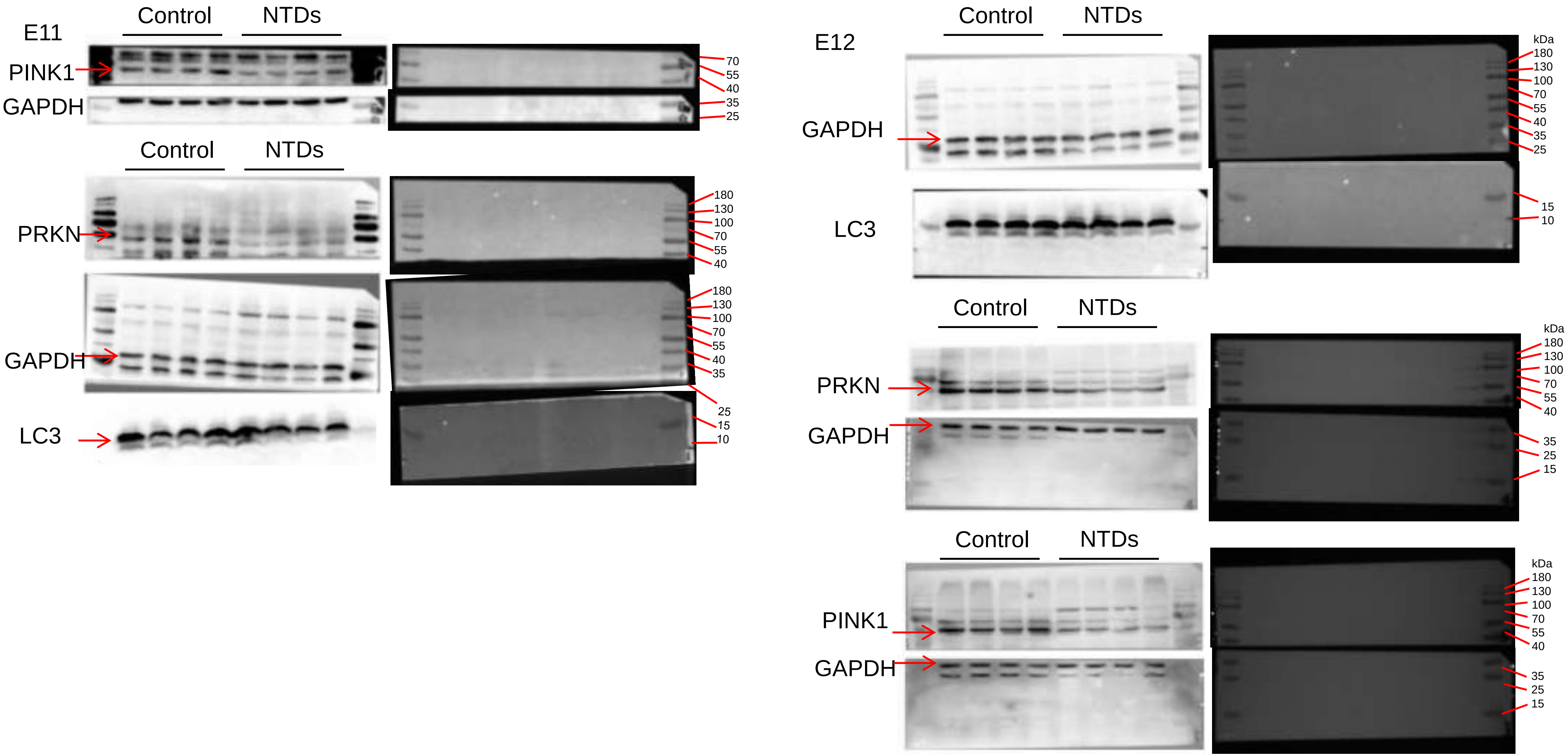
E11
Control
NTDs
PINK1
GAPDH
70
55
40
35
25
Control
NTDs
PRKN
180
130
100
70
55
40
GAPDH
180
130
100
70
55
40
35
25
LC3
15
10
E12
Control
NTDs
kDa
180
130
100
70
55
40
35
25
GAPDH
LC3
15
10
Control
NTDs
kDa
180
130
100
70
55
40
PRKN
GAPDH
35
25
15
Control
NTDs
kDa
180
130
100
70
55
40
PINK1
GAPDH
35
25
15

**Figure 3 I**

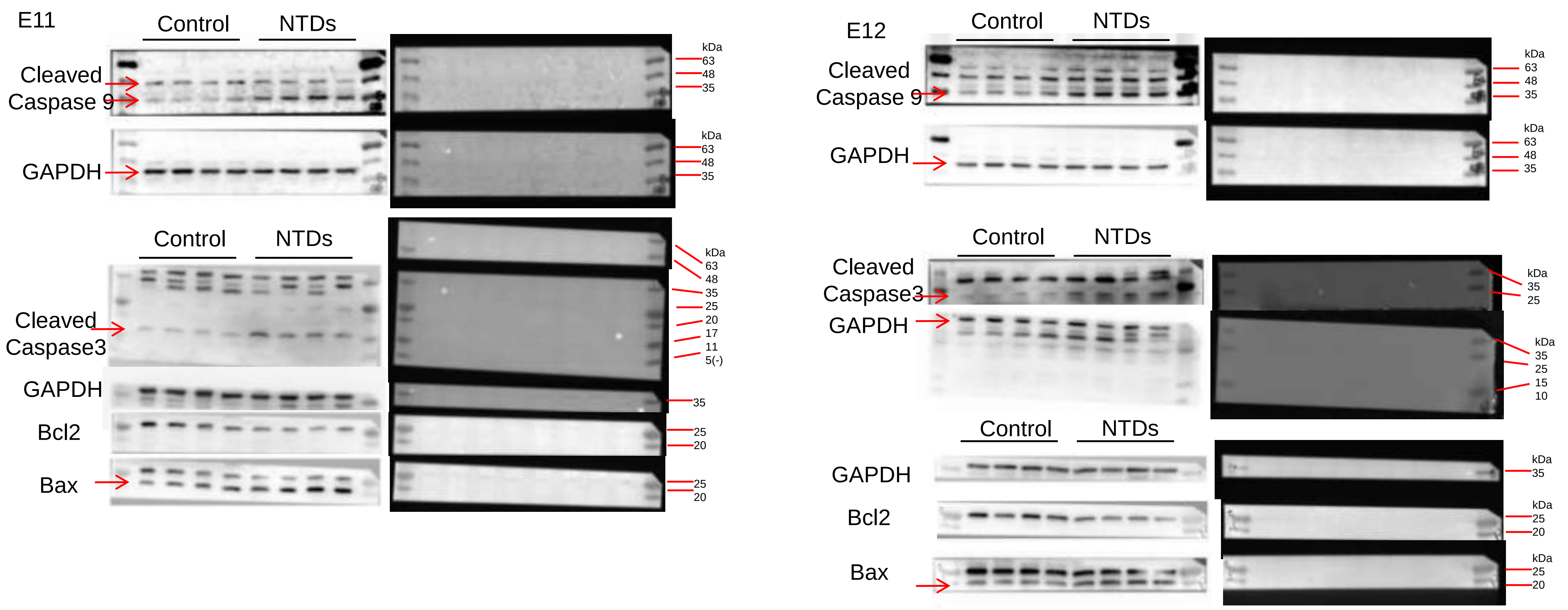
E11
Control
NTDs
Cleaved
Caspase 9
GAPDH
kDa
63
48
35
Cleaved
Caspase3
Bcl2
Bax
25
20
17
11
5(-)
E12
10
15

**Figure 4 A**

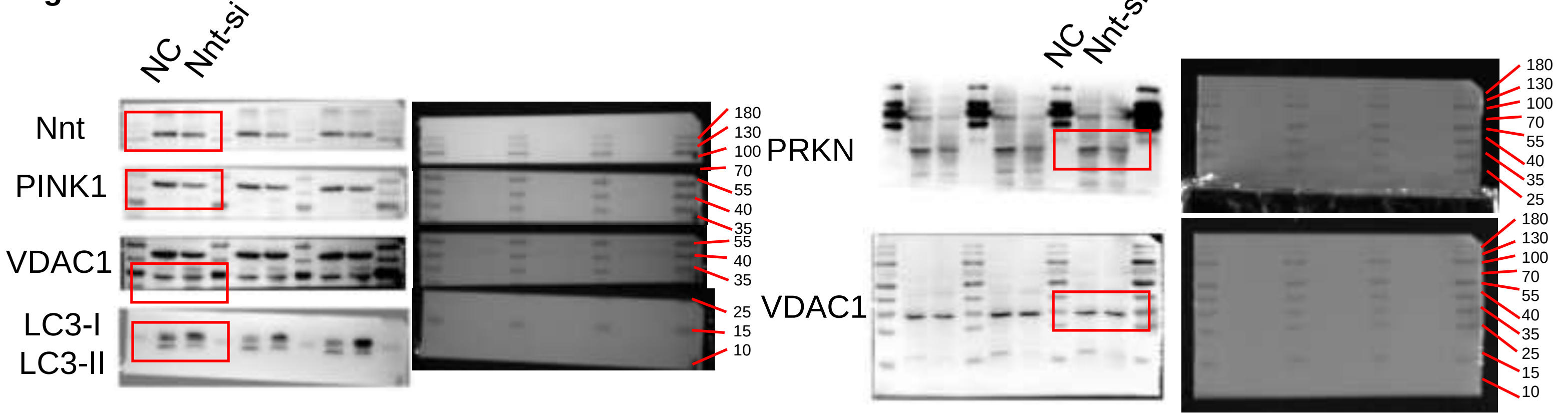


**Figure 4 B**

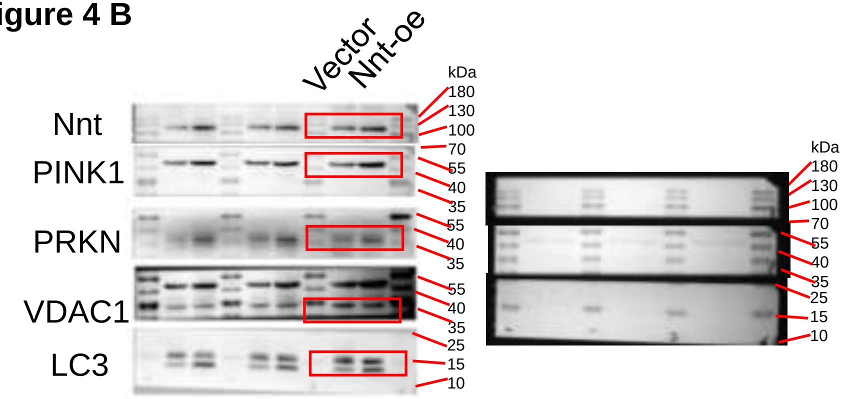


**Figure 4 D**

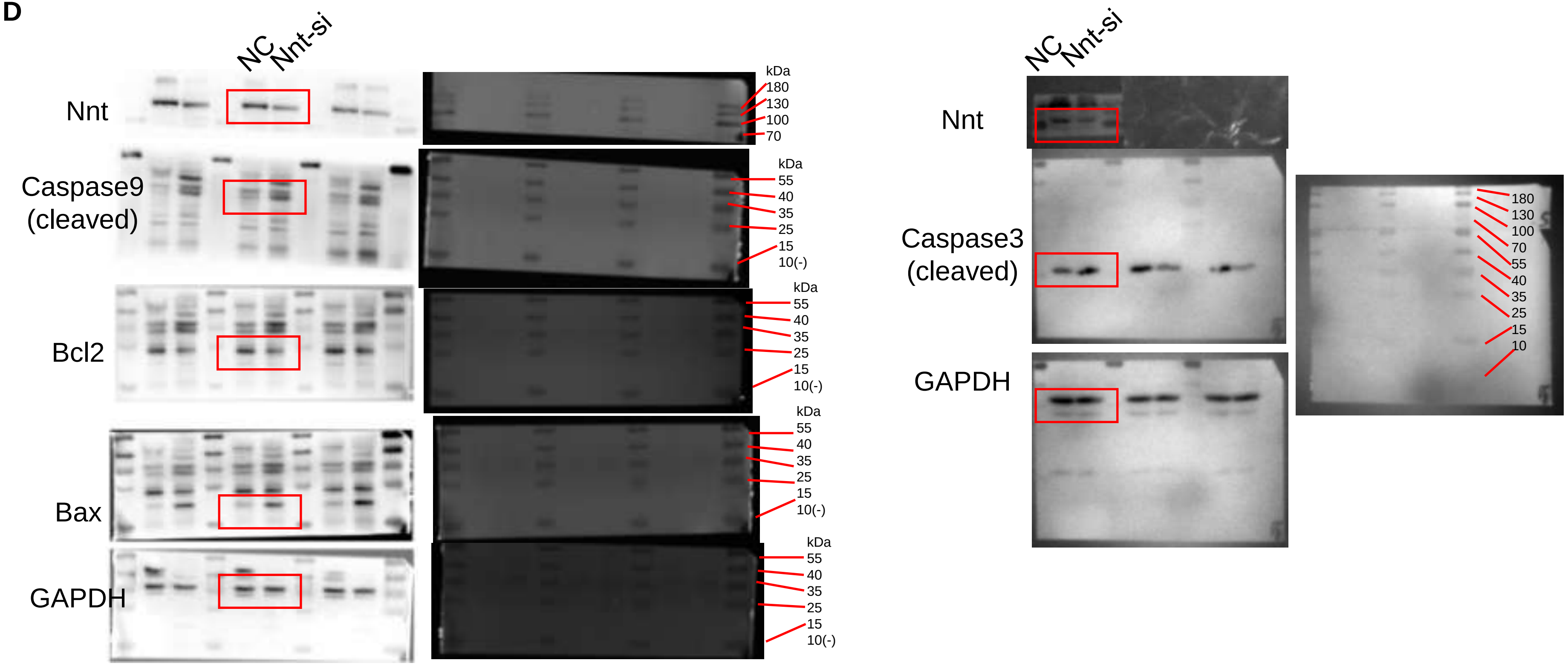

Figure 4 E

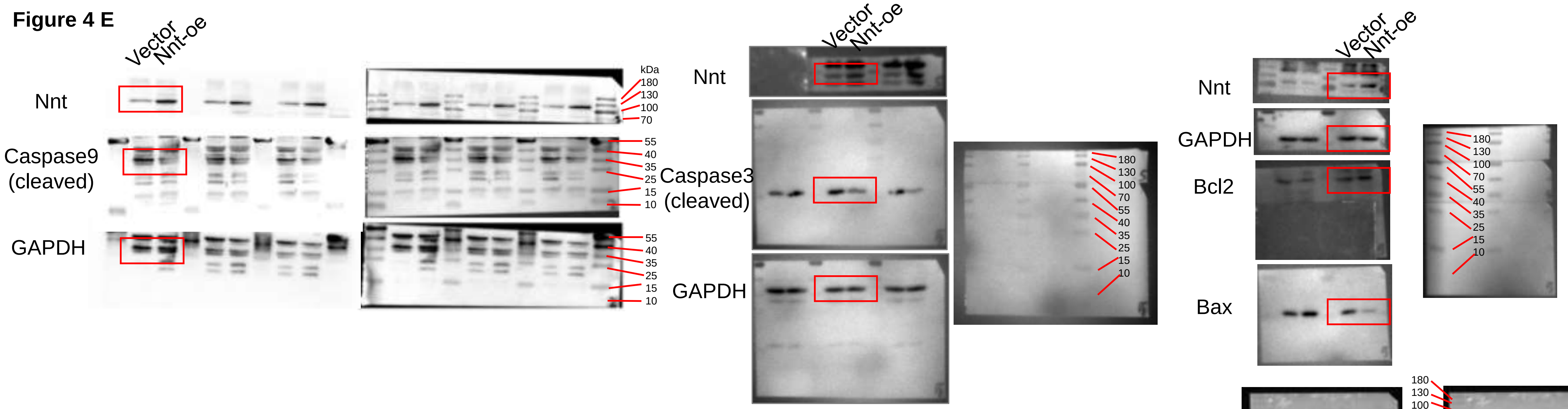


Figure 4 F

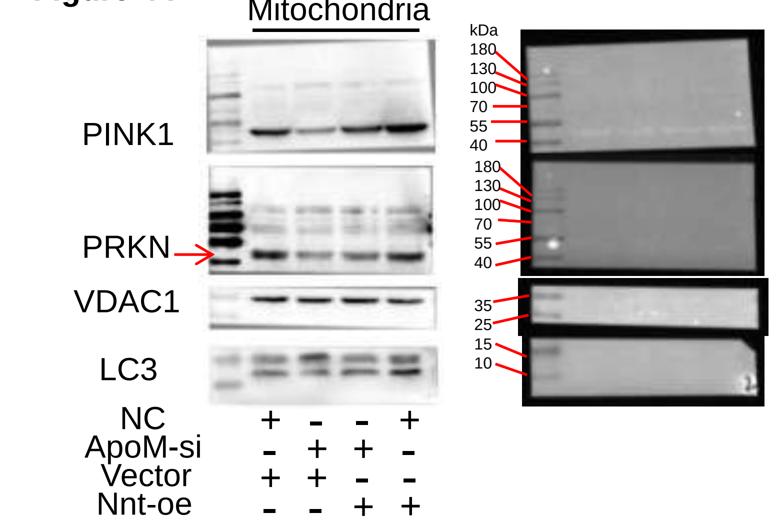


Figure 4 G

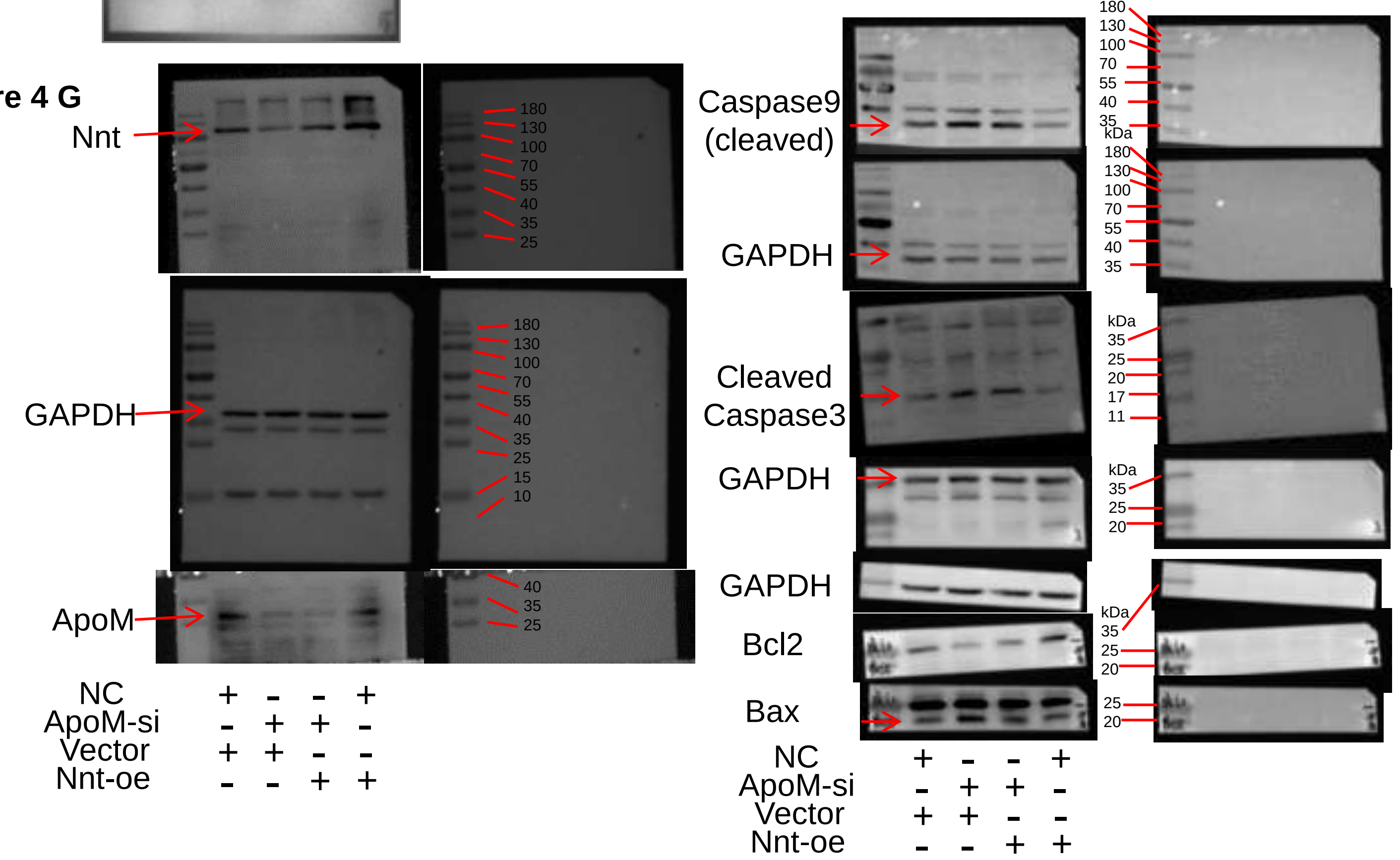

**Figure 5 B**

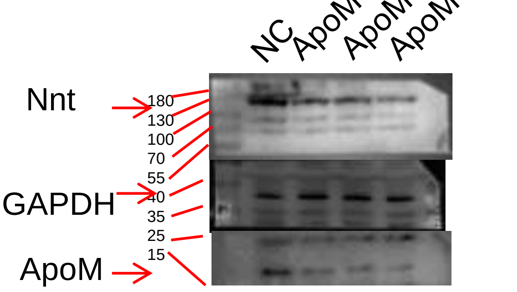


**Figure 5 D**

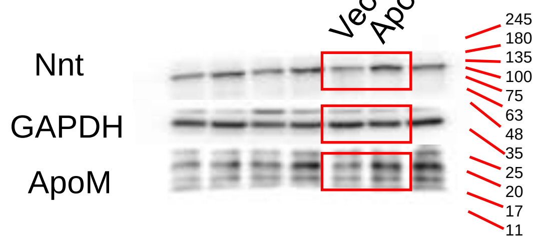


**Figure 5 J**

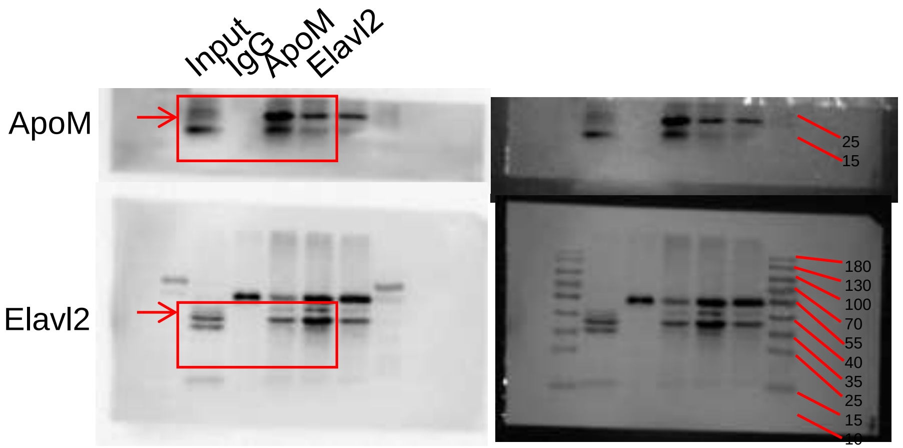


**Figure 5 K**

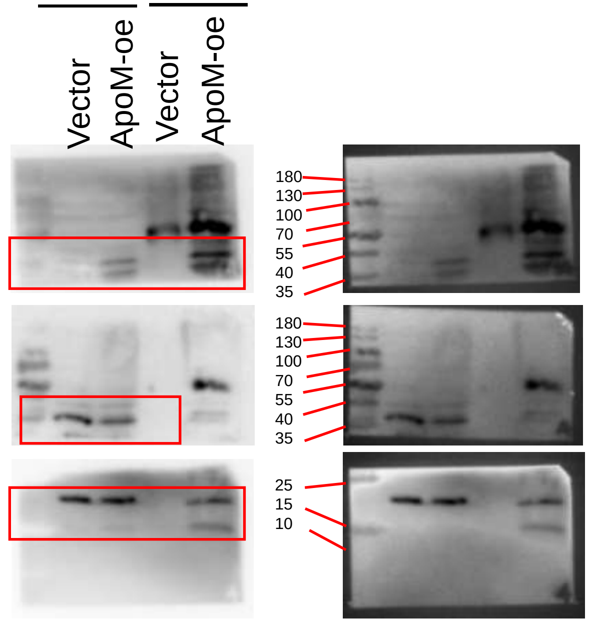


**Figure 5 N**

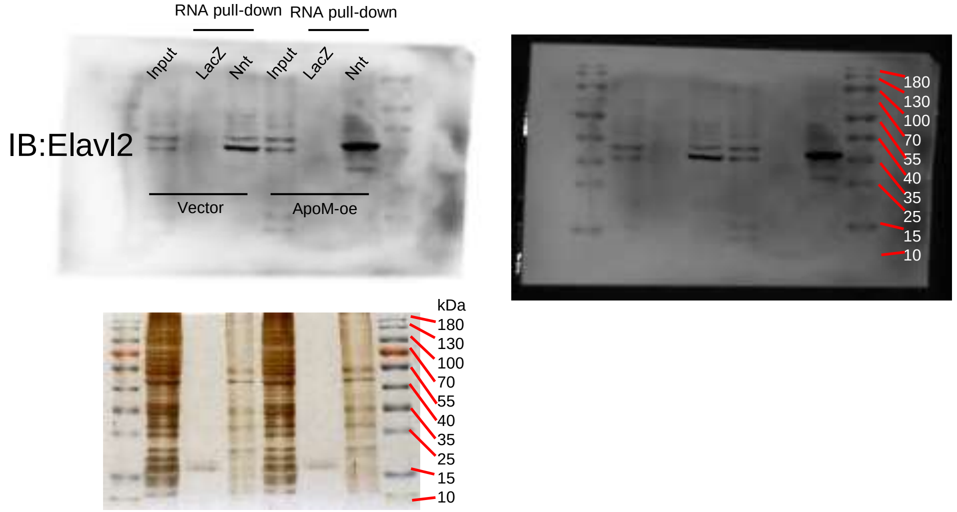


**Figure 5 P-Q**

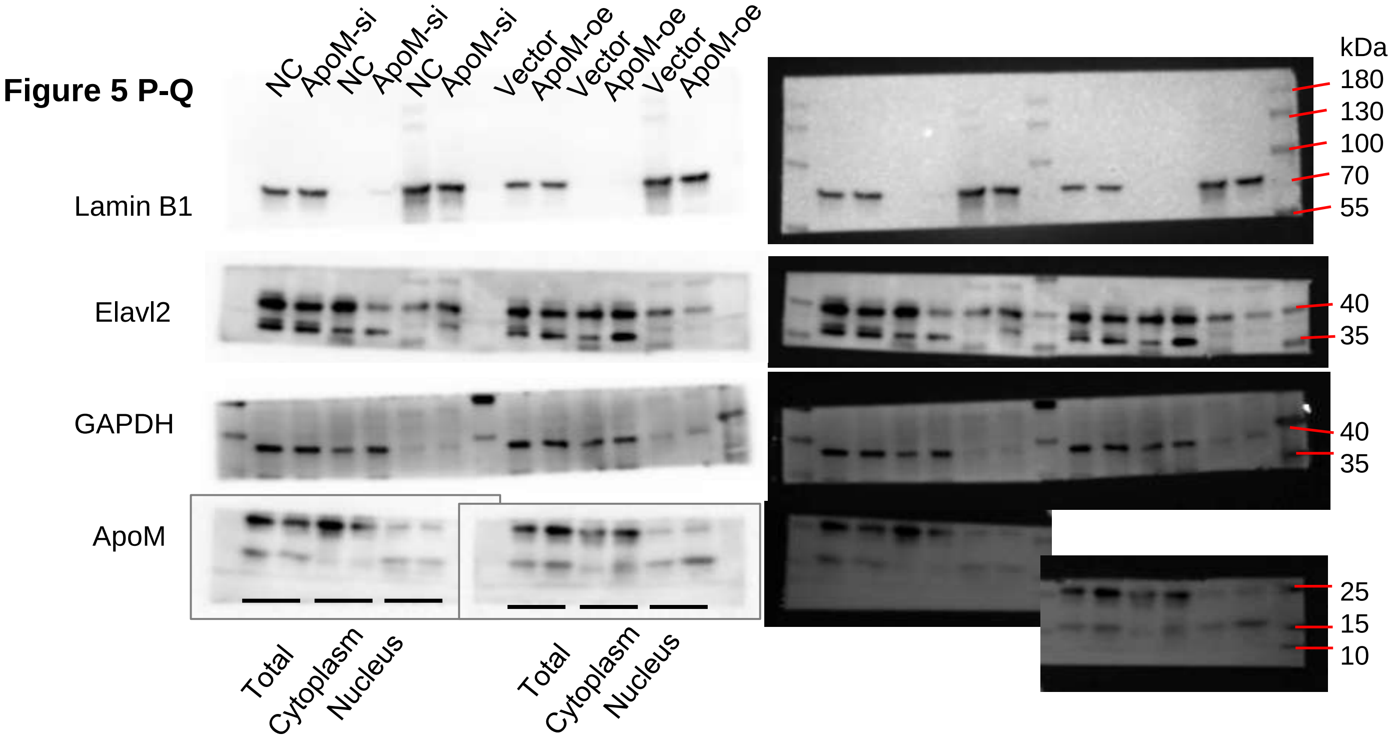

**Figure 5 R**

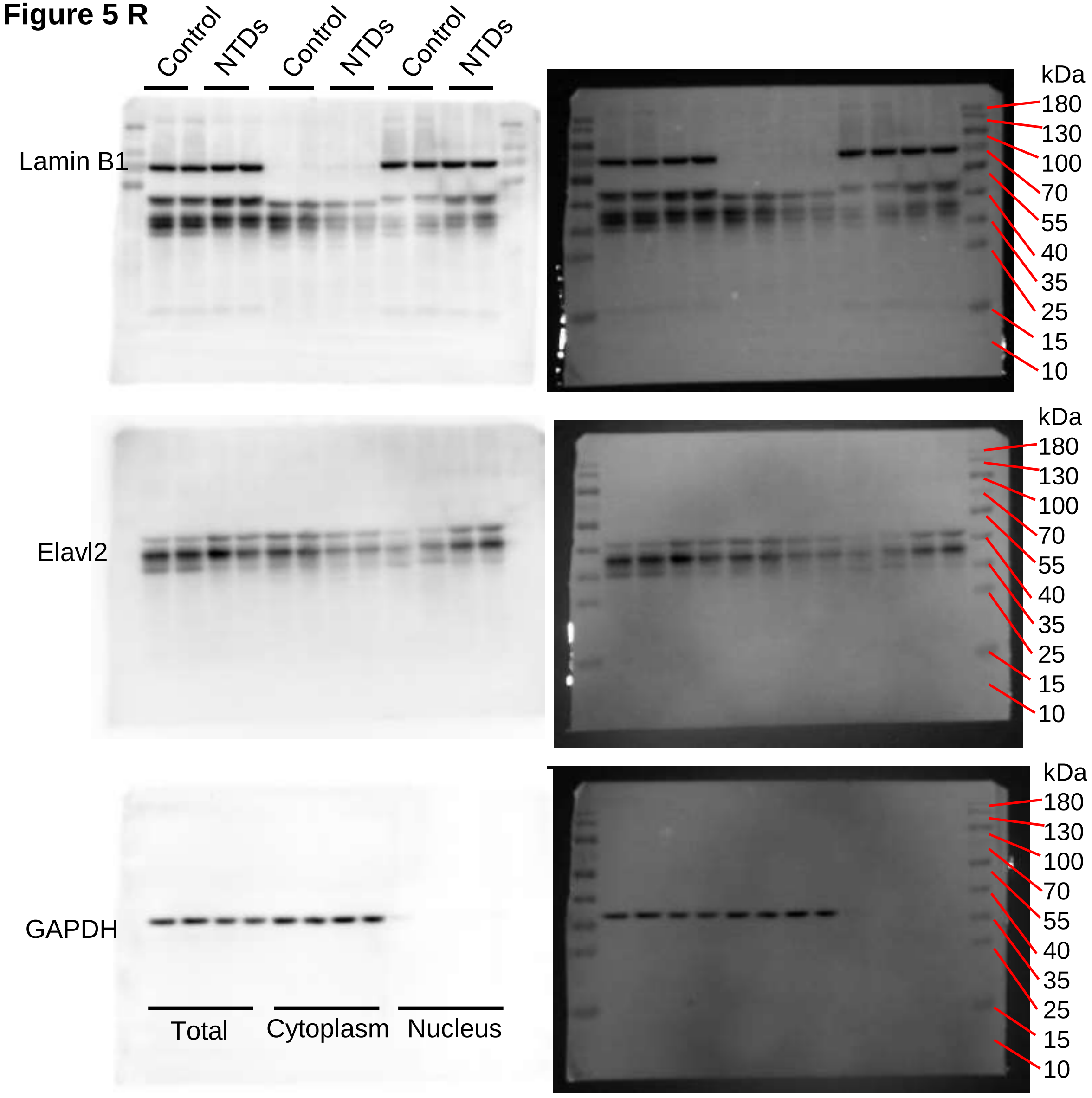
Control
NTDs
Control
NTDs
Control
NTDs
Lamin B1
Elavl2
GAPDH
Total
Cytoplasm
Nucleus
kDa
180
130
100
70
55
40
35
25
15
10
kDa
180
130
100
70
55
40
35
25
15
10
kDa
180
130
100
70
55
40
35
25
15
10

**Figure 6 C**

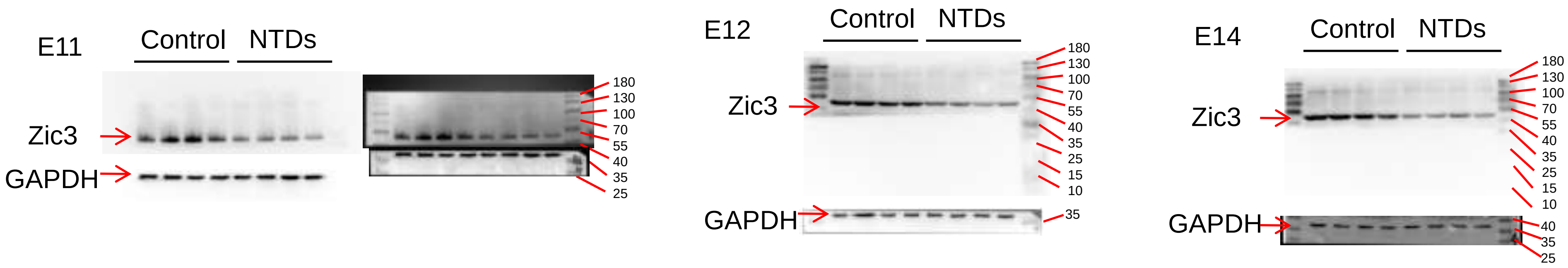


**Figure 6 E**

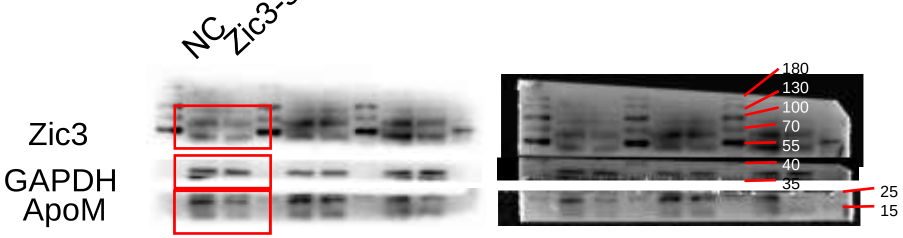


**Figure 6 G**

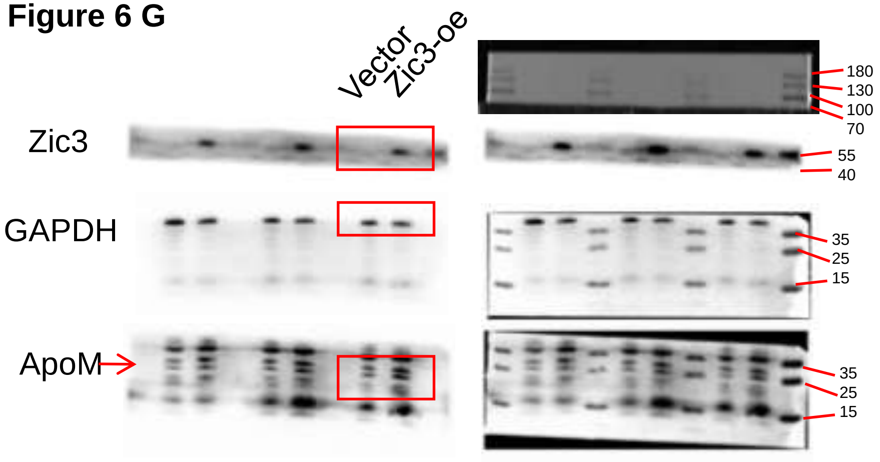

**Figure 7 A**

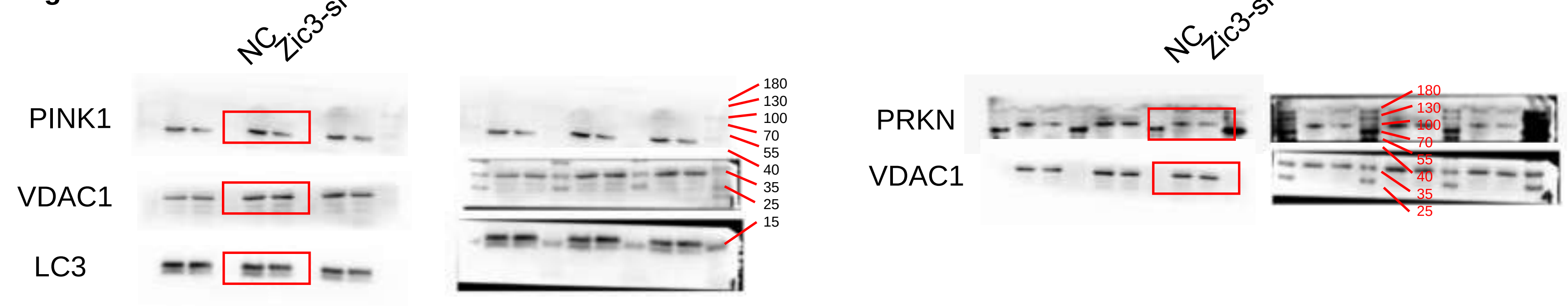


**Figure 7 B**

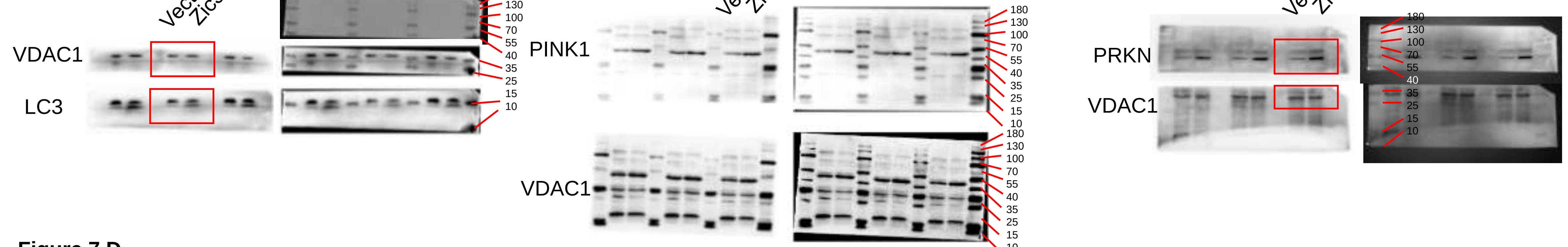


**Figure 7 D**

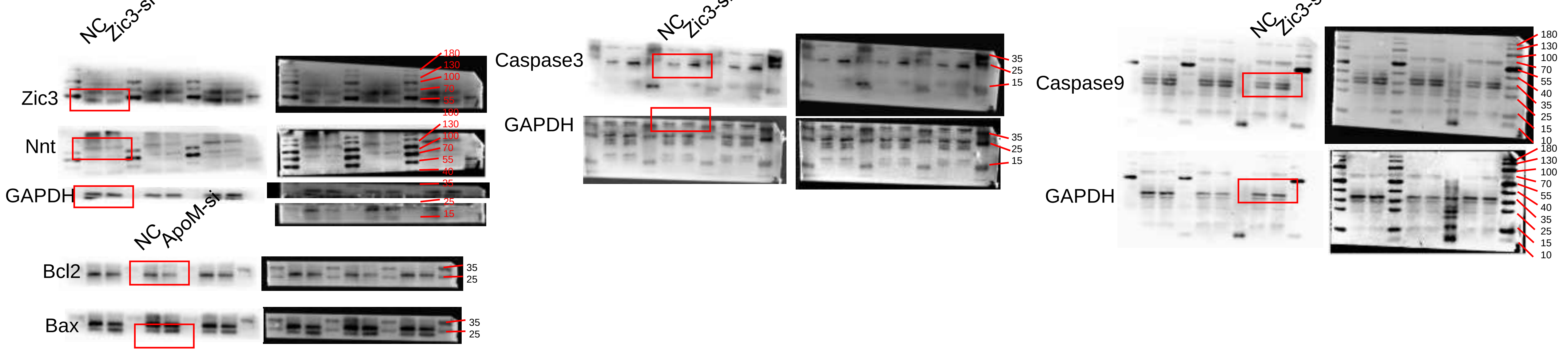

**Figure 7 E**

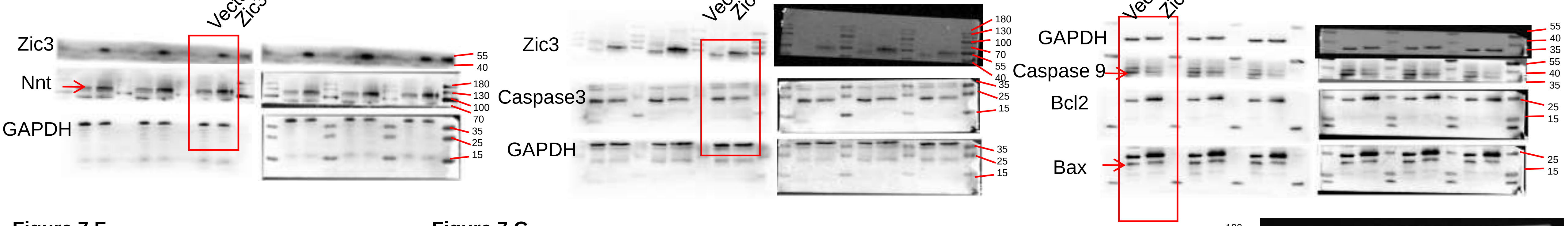


**Figure 7 F**

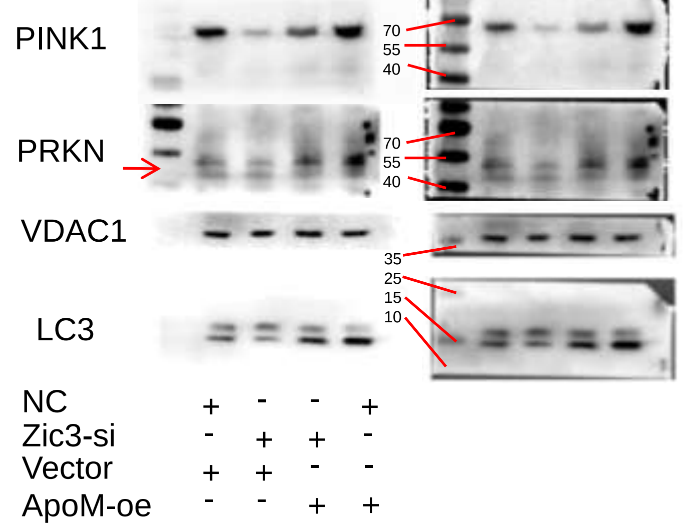


**Figure 7 G**

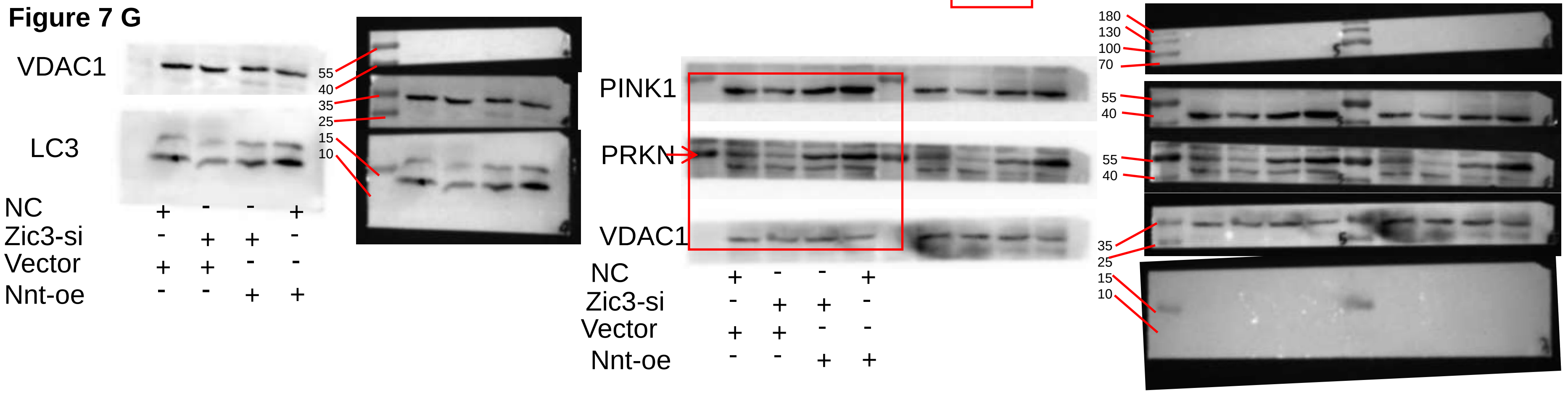

**Figure 7 H**

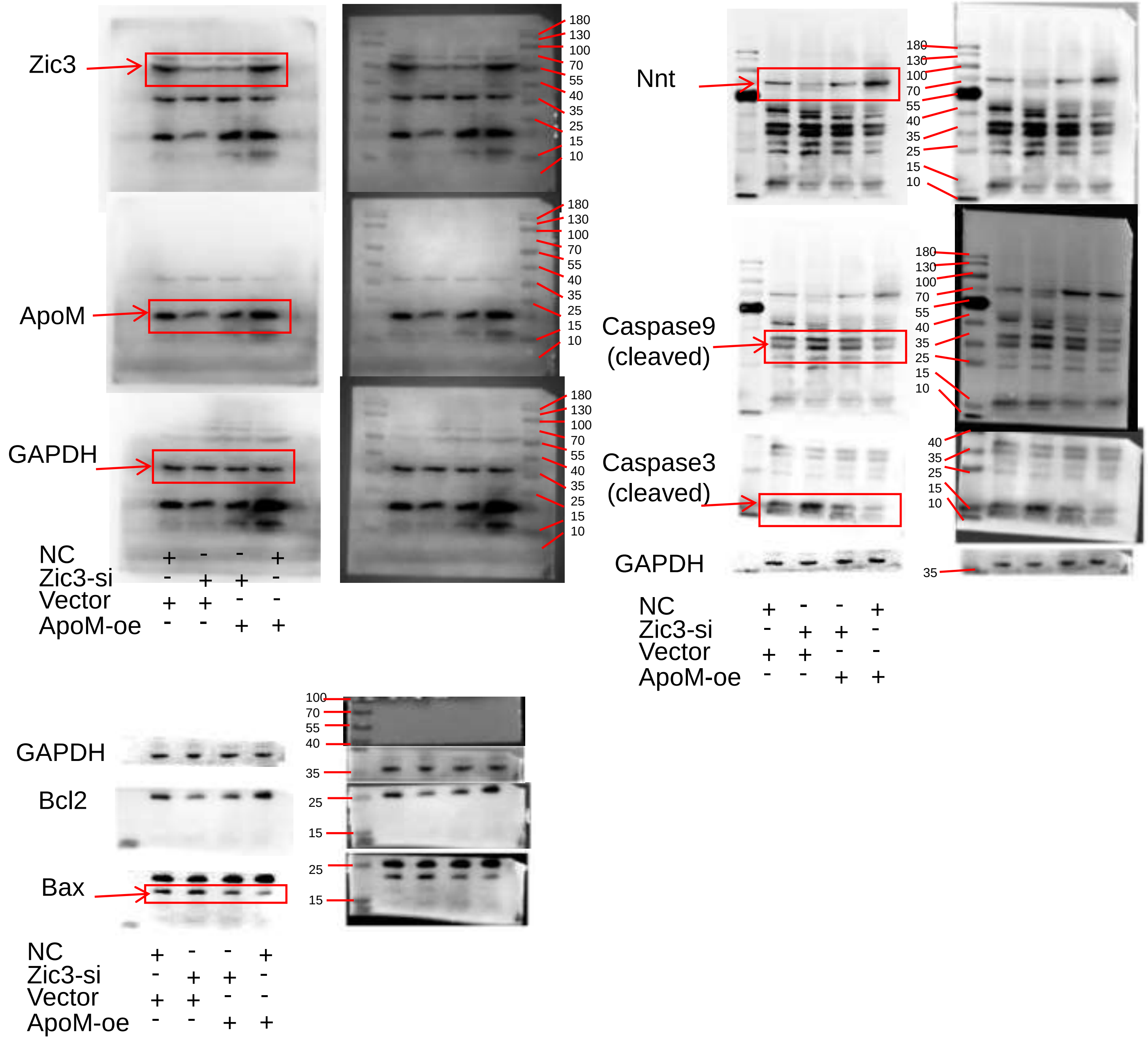


**Figure 7 I**

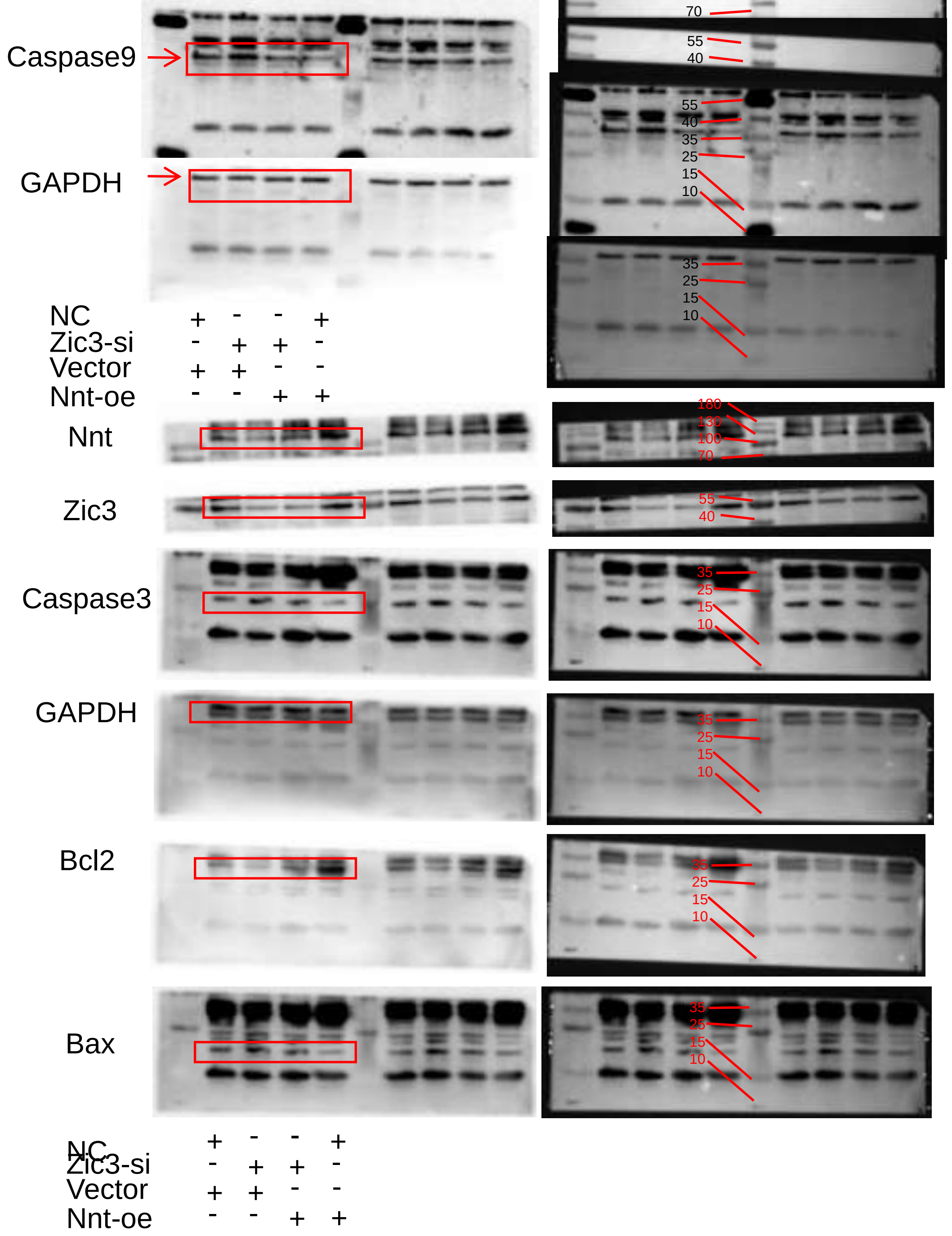

**Figure 8 D**

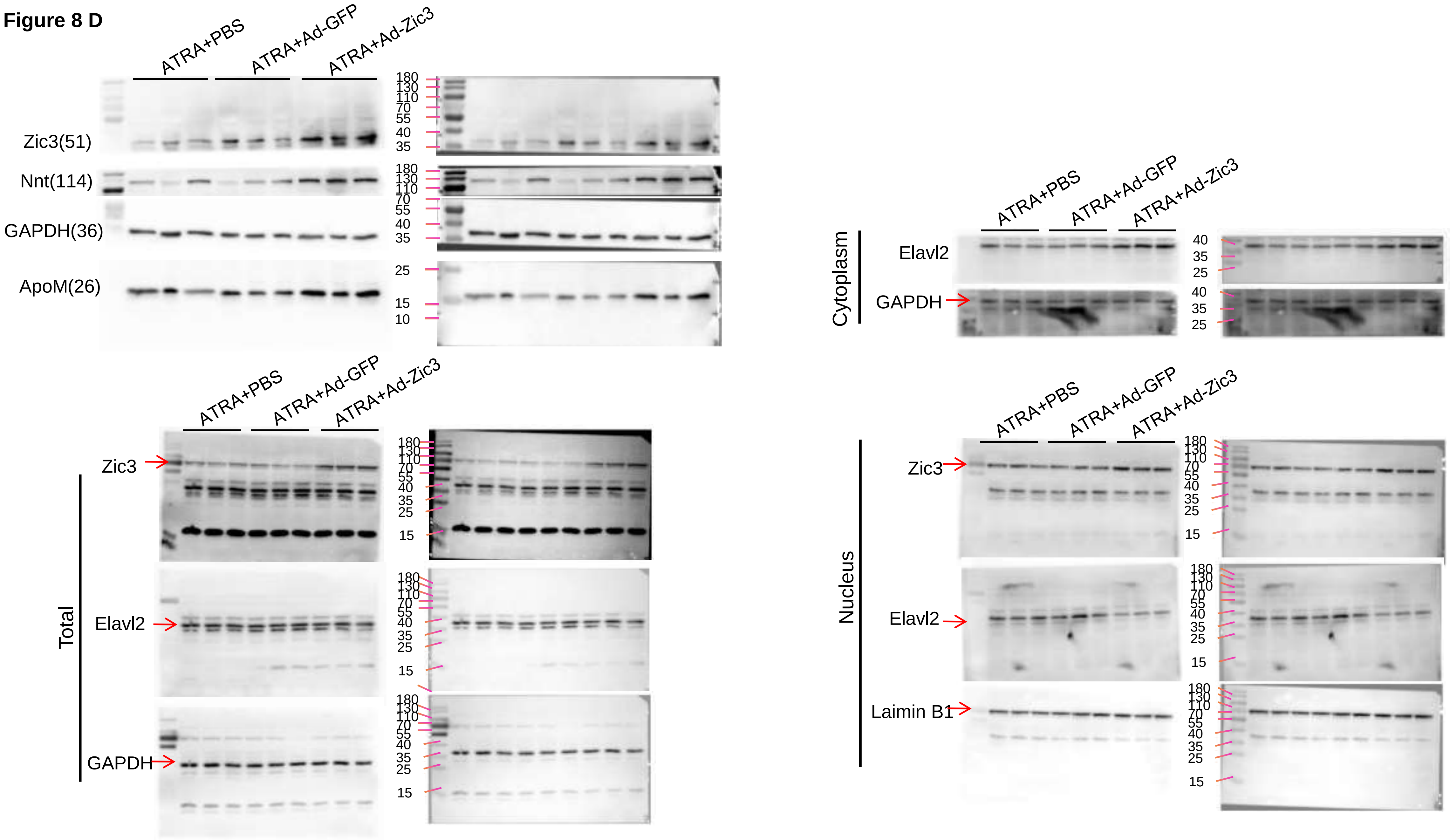
ATRA+PBS
ATRA+Ad-GFP
ATRA+Ad-Zic3
Zic3(51)
Nnt(114)
GAPDH(36)
ApoM(26)
180
130
110
70
55
40
35
25
15
10
Cytoplasm
Elavl2
GAPDH
Total
Zic3
Elavl2
GAPDH
Nucleus
Zic3
Elavl2
Laimin B1

**Figure 8 E**

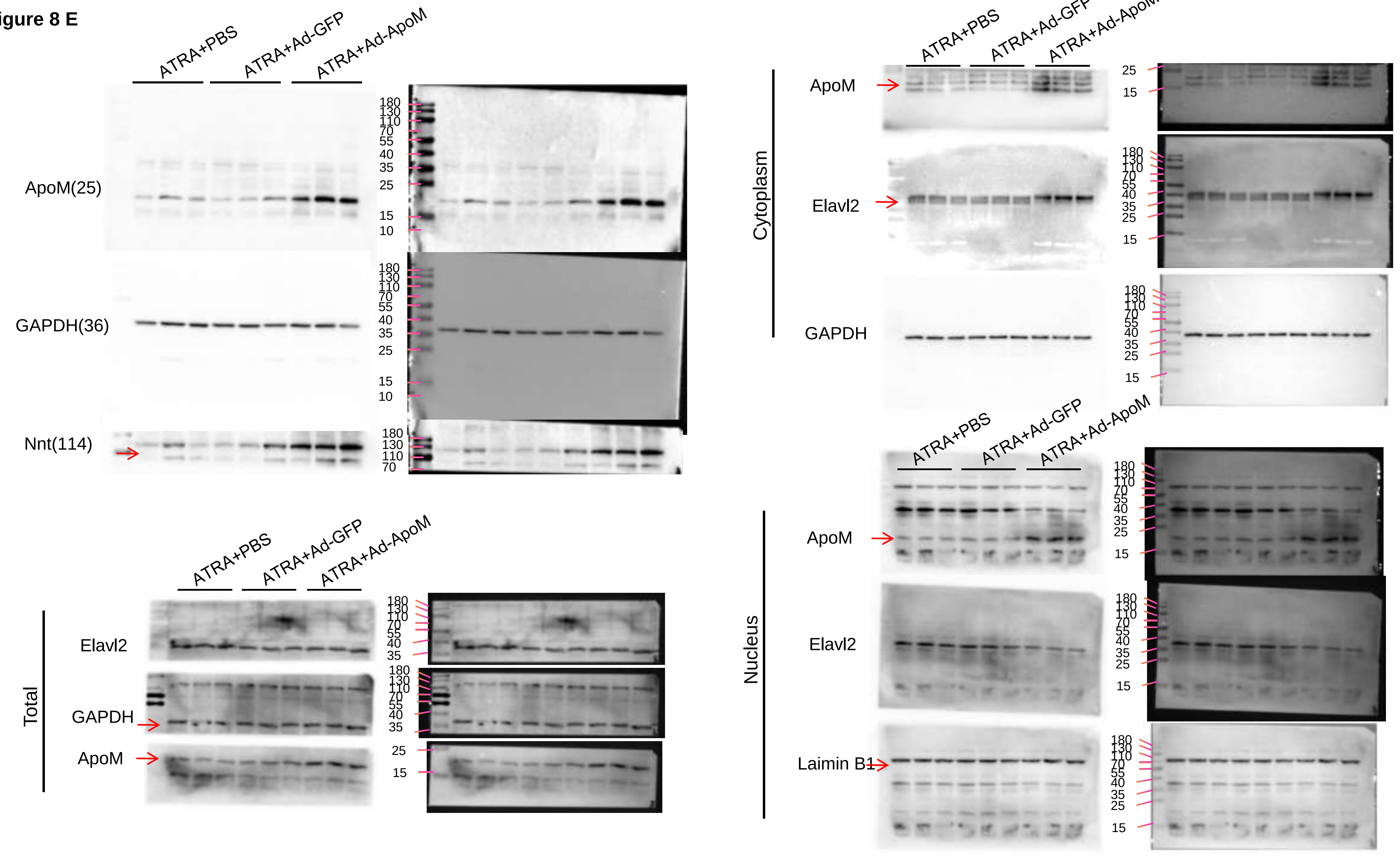
ATRA+PBS
ATRA+Ad-GFP
ATRA+Ad-ApoM
ApoM(25)
GAPDH(36)
Nnt(114)
180
130
110
70
55
40
35
25
15
10
Total
Elavl2
GAPDH
ApoM
Cytoplasm
ApoM
Elavl2
GAPDH
Nucleus
ApoM
Elavl2
Laimin B1

**Figure 8 H**

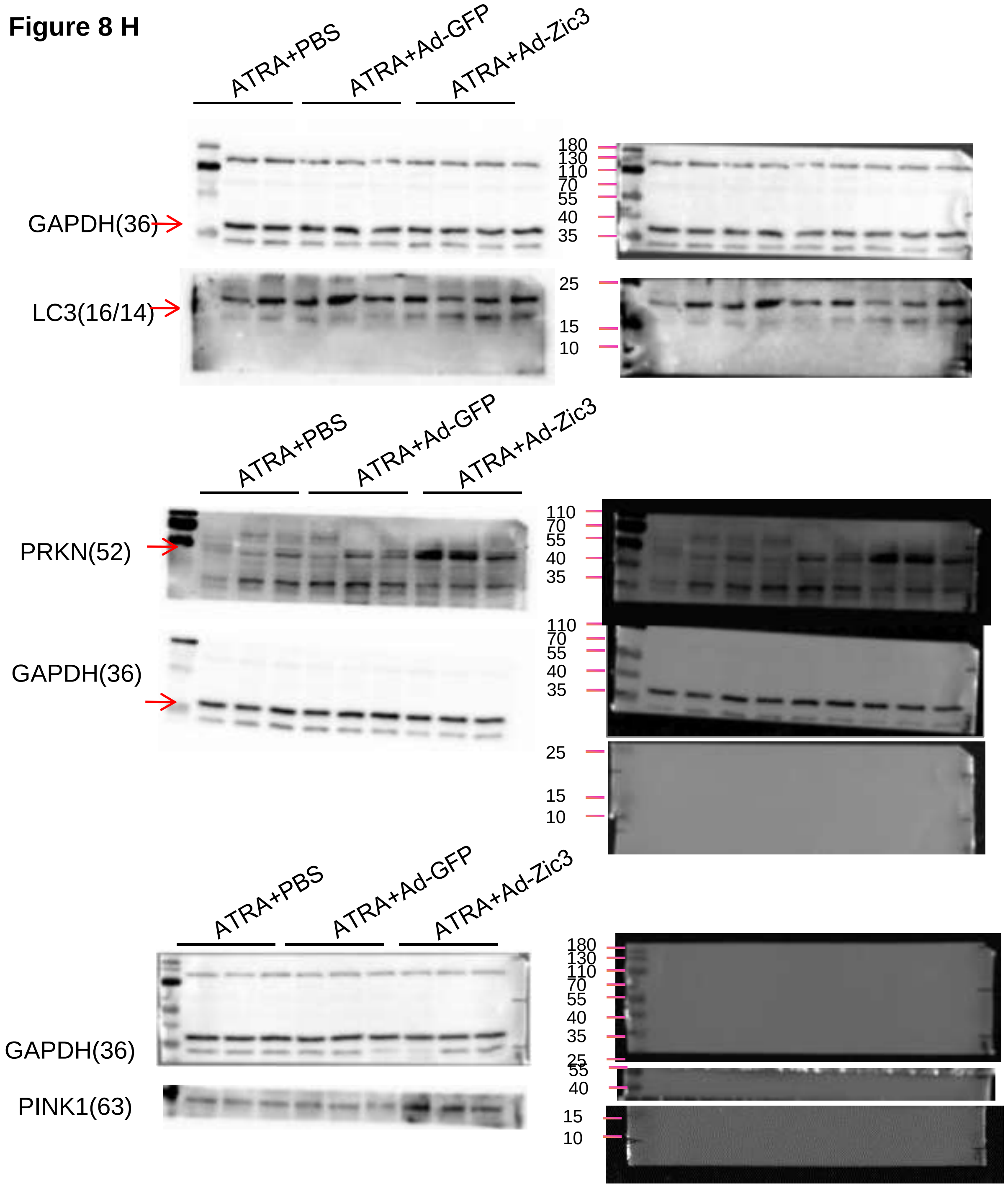


**Figure 8 I**

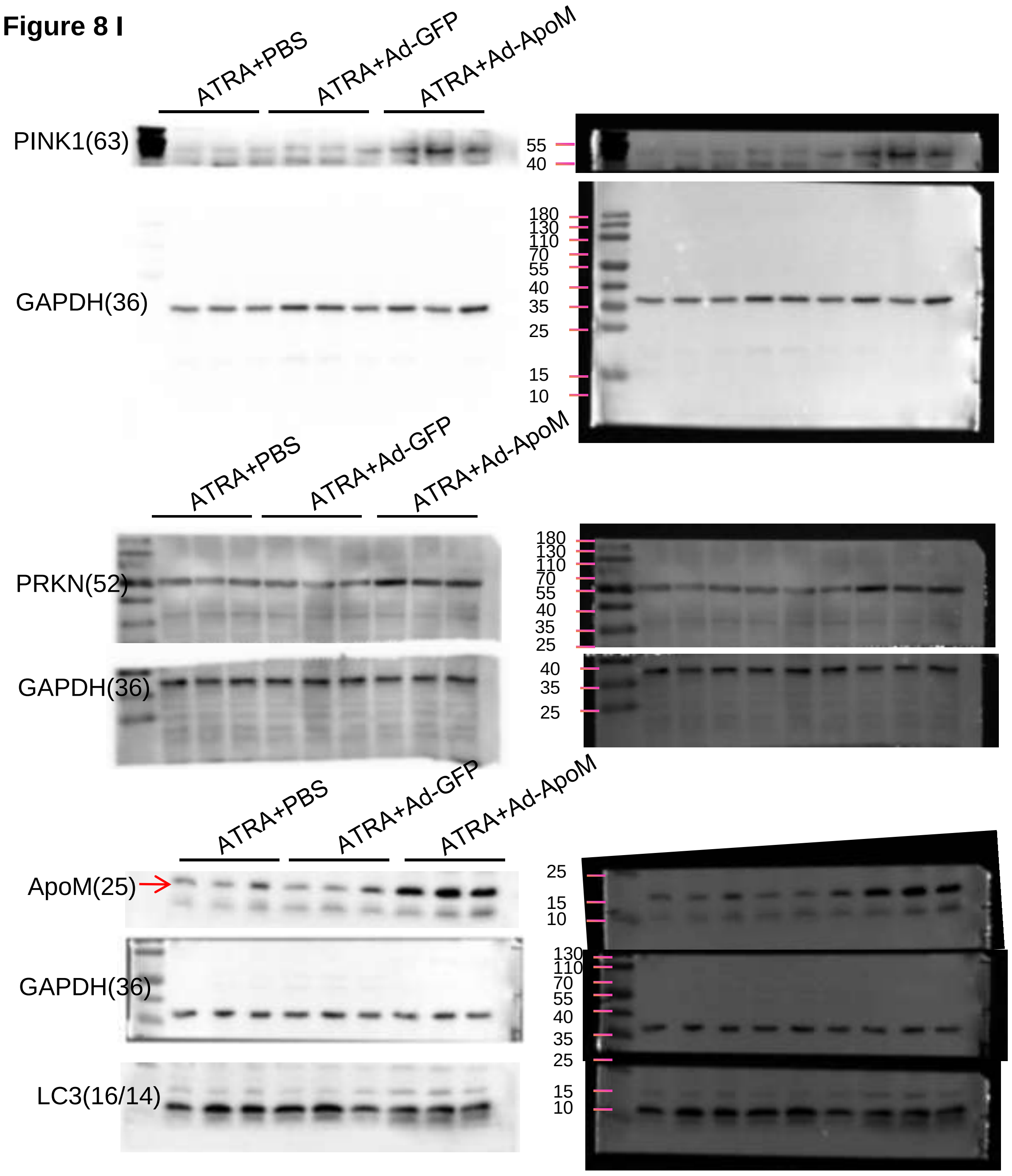

**Figure 8 L**

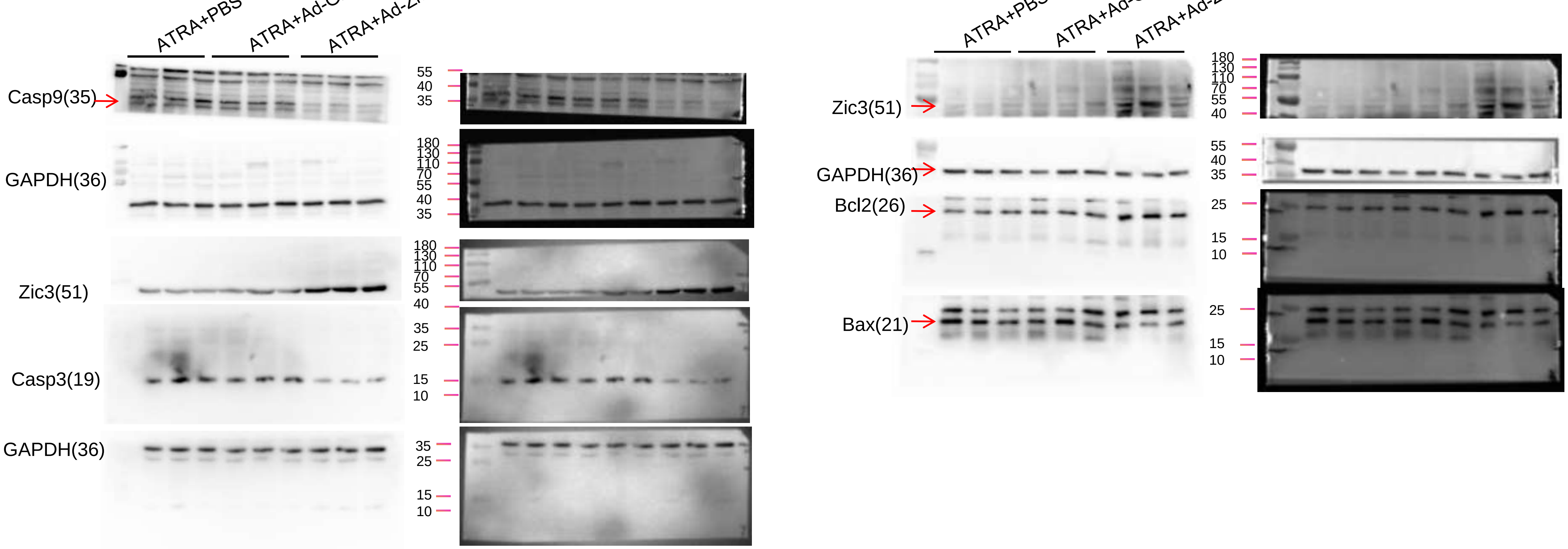
ATRA+PBS
ATRA+Ad-GFP
ATRA+Ad-Zic3
Casp9(35)
GAPDH(36)
Zic3(51)
Casp3(19)
GAPDH(36)
55
40
35
180
130
110
70
55
40
35
180
130
110
70
55
40
35
25
15
10
35
25
15
10
ATRA+PBS
ATRA+Ad-GFP
ATRA+Ad-Zic3
Zic3(51)
GAPDH(36)
Bcl2(26)
Bax(21)
180
130
110
70
55
40
55
40
35
25
15
10
25
15
10

**Figure 8 M**

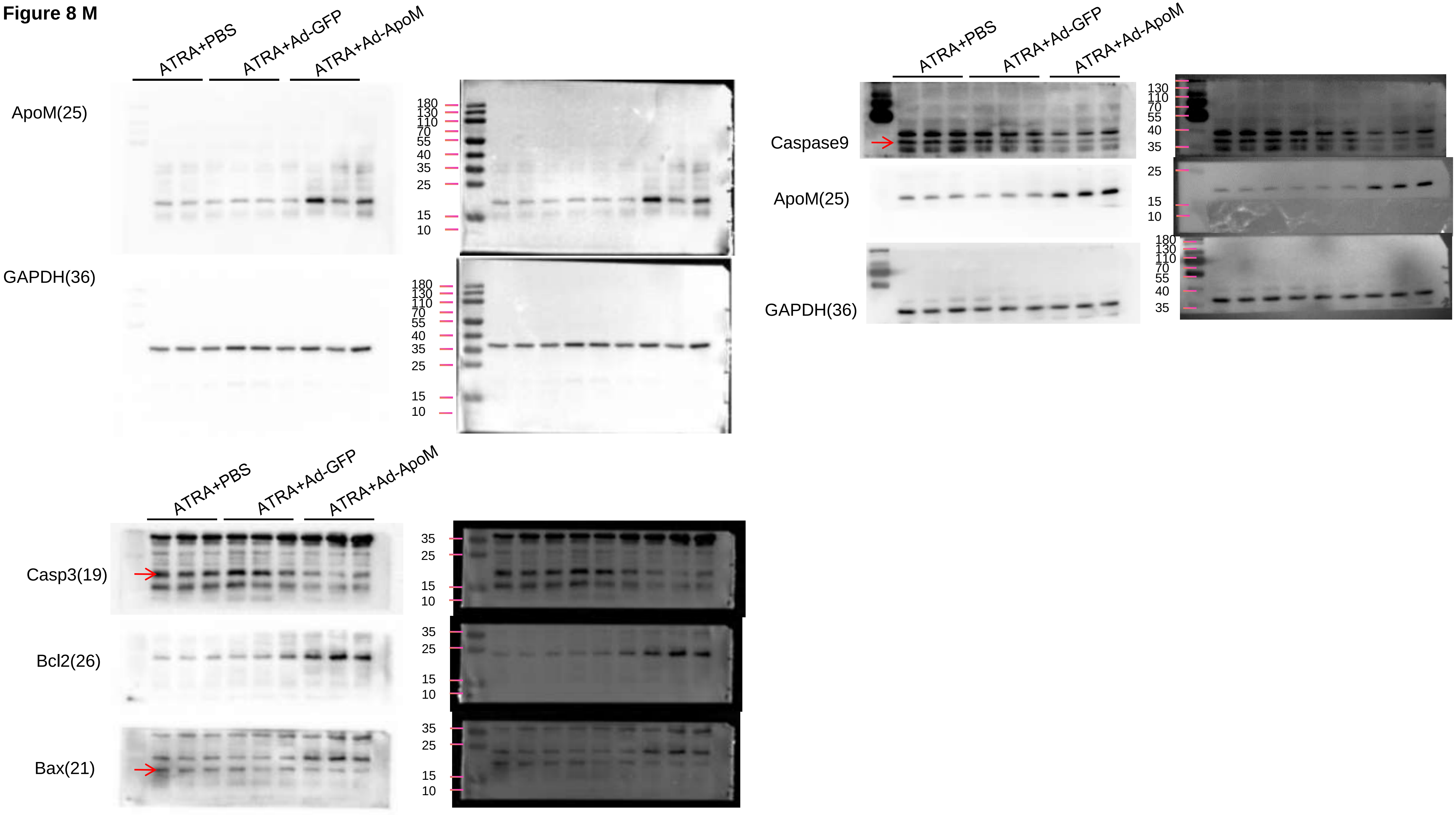
ATRA+PBS
ATRA+Ad-GFP
ATRA+Ad-ApoM
ApoM(25)
GAPDH(36)
Casp3(19)
Bcl2(26)
Bax(21)
Caspase9
180
130
110
70
55
40
35
25
15
10
